# Environmental Factors Affecting the Expression of *pilAB* as Well as the Proteome and Transcriptome of the Grass Endophyte *Azoarcus* sp. Strain BH72

**DOI:** 10.1371/journal.pone.0030421

**Published:** 2012-01-20

**Authors:** Lena Hauberg-Lotte, Hannah Klingenberg, Christian Scharf, Melanie Böhm, Jörg Plessl, Frauke Friedrich, Uwe Völker, Anke Becker, Barbara Reinhold-Hurek

**Affiliations:** 1 University Bremen, Molecular Plant Microbiology, Center for Biomolecular Interactions Bremen, Bremen, Germany; 2 Ernst-Moritz-Arndt-University Greifswald, Interfaculty Institute of Genetics and Functional Genomics, Greifswald, Germany; 3 University of Medicine Greifswald, Department of Otorhinolaryngology, Greifswald, Germany; 4 Institute of Biology III, Faculty of Biology, University of Freiburg, Freiburg, Germany; Cinvestav, Mexico

## Abstract

**Background:**

Bacterial communication is involved in regulation of cellular mechanisms such as metabolic processes, microbe-host interactions or biofilm formation. In the nitrogen-fixing model endophyte of grasses *Azoarcus* sp. strain BH72, known cell-cell signaling systems have not been identified; however, the *pilA* gene encoding the structural protein of type IV pili that are essential for plant colonization appears to be regulated in a population density-dependent manner.

**Methodology/Principal Findings:**

Our data suggest that *pilAB* expression is affected by population density, independent of autoinducers typical for Gram-negative bacteria, likely depending on unknown secreted molecule(s) that can be produced by different bacterial species. We used transcriptomic and proteomic approaches to identify target genes and proteins differentially regulated in conditioned supernatants in comparison to standard growth conditions. Around 8% of the 3992 protein-coding genes of *Azoarcus* sp. and 18% of the detected proteins were differentially regulated. Regulatory proteins and transcription factors among the regulated proteins indicated a complex hierarchy. Differentially regulated genes and proteins were involved in processes such as type IV pili formation and regulation, metal and nutrient transport, energy metabolism, and unknown functions mediated by hypothetical proteins. Four of the newly discovered target genes were further analyzed and in general they showed regulation patterns similar to *pilAB*. The expression of one of them was shown to be induced in plant roots.

**Conclusion/Significance:**

This study is the first global approach to initiate characterization of cell density-dependent gene regulation mediated by soluble molecule(s) in the model endophyte *Azoarcus* sp. strain BH72. Our data suggest that the putative signaling molecule(s) are also produced by other *Proteobacteria* and might thus be used for interspecies communication. This study provides the foundation for the development of robust reporter systems for *Azoarcus* sp. to analyze mechanisms and molecules involved in the population-dependent gene expression in this endophyte in future.

## Introduction

Quorum sensing (QS) is a regulatory mechanism operating in response to cell density. This cell-to-cell communication system is widespread in bacteria and involves the production and detection of quorum sensing signal molecules, termed autoinducers, followed by transcriptional gene regulation [1], [2]. In this way bacteria are capable to communicate with each other and partners in their environment via diverse signal molecules to allow concerted activities of a community. In general, Gram-negative bacteria widely use *N*-acyl-homoserine lactones (AHL) as quorum sensing signal molecules, whereas Gram-positives communicate via peptide-based quorum sensing systems. However, several other autoinducers have been discovered such as the quinolone signal from *Pseudomonas* sp. (PQS, [3]), Autoinducer-2 (AI-2, [4]) for several bacteria or diffusible signal factors (DSF, [5], [6], [7]) for *Xanthomonas campestris*, *Xylella fastidiosa* and *Burkholderia cenocepacia*.

Quorum sensing is important to regulate a wide range of cellular processes such as biofilm formation, virulence, exopolysaccharide production, twitching and swarming motility or siderophore production [8]. Moreover, plant-microbe interactions in several plant pathogens (*Erwinia carotovora*, *Burkholderia pseudomallei*), plant-associated bacteria (*Pseudomonas putida*) or symbionts (*Sinorhizobium meliloti*) are regulated in a cell density-dependent manner [9], [10], [11], [12].

The betaproteobacterium *Azoarcus* sp. BH72 is a nitrogen-fixing model endophyte of grasses [13], [14], able to colonize rice roots under laboratory conditions [15], [16]. Molecular mechanisms of interactions between grass endophytes and their hosts are as yet largely unknown [17]. Surprisingly, there is no evidence for genes encoding the AHL autoinducer synthesis protein or corresponding receptor proteins, and genes coding for the AI-2 synthase are also lacking [13]. Moreover, cross-streak experiments with different AHL sensor strains showed no response to *Azoarcus* sp. BH72 [13], suggesting that this widespread communication system is not used.

Here we describe for the first time evidence for gene regulation in a population density-dependent manner in *Azoarcus* sp. BH72, exemplified by *pilAB* expression. The *pilAB* operon is essential for type IV pilus formation, where *pilA* is coding for a short prepilin [18]. Type IV pili are one of the few known determinants for endophytic establishment of diazotrophic grass endophytes: in *Azoarcus* sp. BH72 they are required for the efficient colonization of plant and fungal host surfaces [18], and also for endophytic spreading in rice roots [19]. Thus, a study on the transcriptional regulation of *pilAB* genes and the respective regulon might reveal further genes required for the plant-microbe interaction.

Our data suggest that this population density-dependent regulation is likely to depend on unknown secreted molecule(s) present in conditioned supernatant and affects expression globally, as evidenced by transcriptome microarray experiments and comparative two-dimensional gel electrophoresis. Our data provide the foundation for the development of robust reporter systems for *Azoarcus* sp. to analyze mechanisms and molecules involved in the population-dependent gene expression in this endophyte in future.

## Results

### Expression of the pilin genes *pilAB* is affected by PilRS and regulated in a population density-dependent manner

The co-transcribed genes *pilA* and *pilB* are crucial for the formation of type IV pili [18]. Upstream of *pilAB*, the two component regulatory system PilSR is encoded [13], with PilR being a transcriptional regulator that likely activates *pilAB* expression [20] upon phosphorylation by PilS or another sensor kinase. Examination of the nucleotide sequence upstream of *pilA* [18] revealed a putative promoter sequence for an alternative sigma factor, σ^54^ (RpoN1 or RpoN2). To determine the transcriptional start site of *pilAB*, primer extension analysis was performed (Figure S1A). The signals indicated a transcriptional start site at the G12 downstream of the putative RpoN-dependent promoter, confirming the utilization of this promoter sequence.

**Figure 1:**
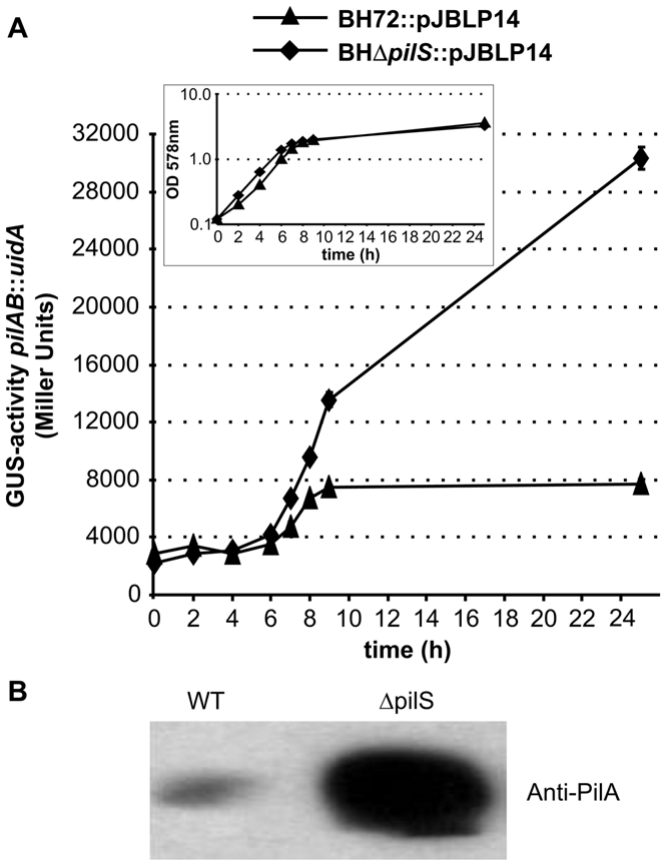
Population density-dependent expression of *pilAB* of *Azoarcus* sp. (A) Population density-dependent induction of *pilAB* gene expression in *Azoarcus* sp. BH72 wild type background and BHΔ*pilS* mutant background. Cultures were grown in liquid aerobic culture (VM-Ethanol), and samples were taken at certain time points to measure β-glucuronidase activity (*pilAB*::*uidA*-fusion, left axis). The optical densities at these time points are shown in the inlay. The results are representative of three independent experiments. Error bars indicate standard deviations. Increase of the expression levels at exponential in comparison to stationary growth phase (last time point) was significant for wild type (BH72::pJBLP14, black triangles, *P*<0.001), and highly significant for *pilS* mutant cells (BHD*pilS*::pJBLP14, black squares, *P*<0.0001, unpaired t-test). (B) PilA protein abundance in *Azoarcus* sp. BH72 wild type and mutant background. Stationary phase cultures (OD 2.5) of *Azoarcus* wild type (wt) and Δ*pilS* mutant cells were compared. Western blot of whole-cell protein extracts with antiserum against PilA. Equal amounts of protein were loaded (8 µg).

In order to analyze the expression level of *pilAB* by transcriptional reporter gene fusions independent of plasmid copy numbers, we constructed a chromosomal *pilAB*::*uidA* fusion strain (BH72::pJBLP14) (Table S1). In the wild type in complex medium (VM-ethanol), the expression increased significantly (2.6-fold, *P*<0.0001) at high population density (Figure 1A, first and last time point). In addition to cell density, conditions of carbon starvation affected *pilAB*::*uidA* expression. GUS activity of cells from a pre-culture in complex medium on ethanol (VM) did not change significantly after transfer to synthetic medium with or without carbon source (Figure S1B); however after one hour of aerobic incubation, expression increased almost 2-fold (1.85, *P*<0.0001) without C-source, but did not change when a carbon source was present (potassium malate).

To elucidate whether the sensor kinase PilS affected the expression pattern, an in-frame *pilS* deletion was constructed that was not likely to cause polar effects (Supporting Information); this mutant *Azoarcus* sp. BHΔ*pilS*::pJBLP14 carried the same chromosomal *pilAB*::*uidA* fusion as above. The GUS activity was not abolished in the *pilS* deletion mutant, in contrast the expression increased more strongly at high cell densities, 10- to 11-fold (*P*<0.0001) (Figure 1A, first and last time point). A similar response was observed for GFP fluorescence as obtained by experiments with *gfp* reporter strains (data not shown). This suggested that PilS had a strong negative effect on the *pilAB* expression under these conditions. The result was confirmed by Western blot analysis; immunodetection of PilA showed a strong increase in whole-cell pilin abundance in the Δ*pilS* mutant in comparison to wild type in the stationary phase (Figure 1B).

In contrast, the induction of *pilAB* expression upon carbon starvation was abolished in the *pilS* deletion (Figure S1B). Thus, PilS appeared to be involved in sensing the absence of carbon sources in the medium, but seemed to act negatively on density-dependent *pilAB* induction.

To analyze the role of PilS further, a point mutant BH*pilS*M was generated in which the conserved histidine residue in the HisKA-domain that was putatively required for phosphorylation was exchanged against arginine (His314Arg) to prevent phosphorylation. Autophosphorylation of an overexpressed truncated PilS variant lacking the transmembrane helices occurred *in vitro* in presence of radiolabeled ATP, while the point mutant indeed failed to be phosphorylated (not shown). The *Azoarcus* mutant BH*pilS*M carrying the chromosomally integrated *pilAB*::*uidA* fusion (pJBLP14) showed an approximately 4-fold decrease in expression in comparison to wild type at early exponential phase (1230±320 Miller units or 4615±520 Miller units, respectively, *P*<0,0001, unpaired t-Test), a ratio which was retained also in stationary growth phase. This suggested that PilS might act as bifunctional sensor kinase acting negatively on PilR phosphorylation and *pilAB* expression when not phosphorylated.

Population density-dependent induction suggested that pilin gene expression in *Azoarcus* sp. strain BH72 might be under the control of “quorum sensing”-like mechanisms. Therefore, we tested whether cell-free supernatants of stationary phase cultures (conditioned supernatants) could induce pilin gene expression in exponentially grown test cells at low cell densities. To exclude putative effects of carbon starvation, ethanol was added to the supernatants. An elevated expression was not observed in the wild type background, where *pilAB* expression was not significantly induced (1.2-fold). However, in the Δ*pilS* background the expression of *pilAB*::*uidA* increased: after one hour of incubation with conditioned supernatant, the GUS activity was almost unchanged (1.3 fold±0.22), whereas an increase was detected after two hours (1.6 fold±0.06). After four hours, the gene expression of *pilAB* was induced 2.4-fold (±0.32; *P*<0.0001) compared to the negative control with medium. Further incubation with conditioned supernatant did not lead to increased GUS activity (data not shown). Although the induction values were relatively low, this observation suggested that the conditioned culture supernatant from *Azoarcus* sp. BH72 had an impact on the *pilAB* gene expression within few hours, likely due to unknown signal molecule(s) or metabolites accumulating in the bacterial conditioned culture supernatant.

### *pilAB* expression is regulated via molecules different from AHL-based or well-known signaling systems

Based on the results above we developed a supernatant bioassay for *Azoarcus* sp. BH72. Briefly, an aliquot of cells of the reporter strain BHΔ*pilS*::pJBLP14 growing exponentially on liquid VM-ethanol medium was transferred to either fresh VM-ethanol medium, or to conditioned supernatant produced by *Azoarcus* wild type. Conditioned supernatants were supplemented with 3 ml ethanol per liter to overcome possible carbon source depletion during previous growth. The induction of *pilAB* expression was not altered when ethanol was not supplemented (data not shown). After four hours of aerobic incubation, *pilAB*::*uidA* expression of washed cells was quantified in GUS assays. This experimental setup was used for mutant studies below.

The bioinformatic analysis of the *Azoarcus* sp. BH72 genome revealed no evidence for genes encoding proteins for known quorum sensing systems, such as homologues of LuxIR/LasIR or LuxS [13]. Therefore, the genome was screened for predicted protein domains that are relevant in known quorum sensing systems. The proteins with best matches were chosen for further analysis, albeit E-values were rather poor. The conserved hypothetical protein Azo3178 carried an autoinducer synthethase (LasI) [21] domain (Pfam PF00765, E-value 9.40e^−03^); the conserved hypothetical protein Azo1746 a peptidase C39 motif (Pfam PF03412, E-value 5.10e^−01^) typical for ABC-type bacteriocine exporters responsible for the export of signaling peptides [22]; proteins Azo0390 and Azo3379 contained a lactamase B domain (for Azo0390 E-value 2.00e^−29^, for Azo3379 E-value 6.40e^−24^) putatively involved in quinolone (PQS) signaling [23]. All four genes were separately inactivated in strain BH72 by directed plasmid insertional mutagenesis (see Table S1), and the mutants analyzed for production of a *pilAB*-inducing conditioned supernatant. The *pilAB*::*uidA* expression in *Azoarcus* supernatant bioassays was still significantly induced (*P*<0.01) by supernatants of all mutants (Table S2), indicating that autoinducer production was not affected by any of the mutations in the candidate proteins.

To further exclude the influence of AHL on *pilAB* induction, conditioned culture supernatant was extracted with dichloromethane, and the extracts were tested in an AHL plate detection assay and in a supernatant bioassay. The AHL-producing strain *Chromobacterium violaceum* CV017 was used as a positive control. For the AHL detection assay three different AHL monitor strains were used: the GFP-based sensor strains described above, or *C. violaceum* CV026 as a reporter for short chain AHL (C4–C8) and cyclic dipeptides. Only dichloromethane extracts of the positive control strain, *Rhizobium* sp. NGR234 producing AHL (3-oxo-C8-HSL) [24], yielded a strong signal for short chain AHLs in both reporter strains. As expected, the extracts of *Azoarcus* yielded no signals (data not shown).

To test whether the putative signaling molecule is hydrophobic, conditioned supernatants were extracted with dichloromethane and subjected to the *Azoarcus* supernatant bioassay. The inducing effect of the untreated conditioned supernatants, the supernatants after extraction with dichloromethane, and the dichloromethane extract were tested for induction of *pilAB*::*uidA* expression in comparison to fresh medium. Inducing activity was not found in the dichloromethane extract, but remained in the conditioned culture supernatant (Table S2). Thus, in contrast to AHLs and most other known autoinducers, the molecule(s) was apparently not sufficiently hydrophobic to be extracted by the solvent. As gene induction was also retained when the conditioned supernatant was passed through an ultrafiltration membrane (size exclusion 1000 Da), the inducing factor appeared to be a small hydrophilic molecule (hydrophilic signal factor, HSF).

### Transcriptomic studies reveal that around 8.0% of all *Azoarcus* genes are differentially regulated in conditioned supernatant

We designed genome-scale experiments to identify additional genes that might be regulated in a manner similar to *pilAB*, of which some might show higher induction values. Characterization of such genes would be instrumental for further genetic or biochemical studies on a putative cell signaling system in *Azoarcus* sp. strain BH72. A whole genome microarray approach was carried out to compare gene expression under standard growth conditions in comparison to conditioned culture supernatant. Briefly, the change of gene expression in wild type cells was monitored after one hour and four hours incubation of cells at low population density in conditioned supernatants obtained from *Azoarcus* wild type. Genes were considered as being differentially expressed if the differential expression level between the tested conditions was at least 1.8 fold and the *P*-value≤0.05, as for many genes significant values could be obtained already at this low induction value. Expression of 7.7% of all *Azoarcus* sp. BH72 genes was found to be influenced by the conditioned culture supernatant containing the unknown molecule(s). This indicated that density-dependent regulation may be an important, global gene regulation process in this grass endophyte. Which portion of the differentially regulated genes was affected by a putative cell-signaling system or by side effects, as the conditioned culture supernatants were obtained from *Azoarcus* cultures from the stationary growth phase which might contain metabolites or antibiotics, is not clear at this stage. From all regulated genes, the mRNA abundance of 157 genes (3.9%) was increased, whereas 150 genes (3.8%) showed decreased mRNA abundance. Out of the up-regulated genes detected by microarray analysis, 39 genes were found to be activated after one as well as four hours of incubation of *Azoarcus* with conditioned culture supernatant, while 36 or 82 genes were up-regulated only after one or four hours, respectively. From all down-regulated genes, 35 genes showed repression after one and four hours of application, while 25 or 90 were down-regulated only after one or four hours, respectively. The detailed results of all differentially regulated genes detected by the transcriptional profiling are listed in Table S3.

It would be expected that nearly all genes in the same operon show differential gene expression in a microarray approach. Indeed, for a number of gene clusters differential regulation was detected for several genes, four typical clusters being depicted in Figure S2. The *atp*-operon (C), coding for the subunits of ATP synthase, as well as the *nuo*-cluster (A) encoding the NADH-ubiquione oxidoreductase chains were repressed at both time points. The *nap*-genes (D), coding for the subunits of the periplasmic nitrate reductase complex, were activated, although cells were cultivated under aerobic conditions with vigorous shaking, so that secondary effects of oxygen supply are not to be expected. Several genes that were down-regulated encoded ribosomal proteins and proteins involved in general translation processes. Those genes have different localization sites in the *Azoarcus* genome, and only one large cluster (B) is shown. This cluster was delimited with three putative terminator structures, leading to four probable transcripts from which only three were differentially regulated, spanning genes from *azo3390* to *azo3422*, from *azo3425* to *azo3428* as well as from *azo3429* to *azo3431*.

### Comparative proteomic studies support the global gene expression approach

**Figure 2:**
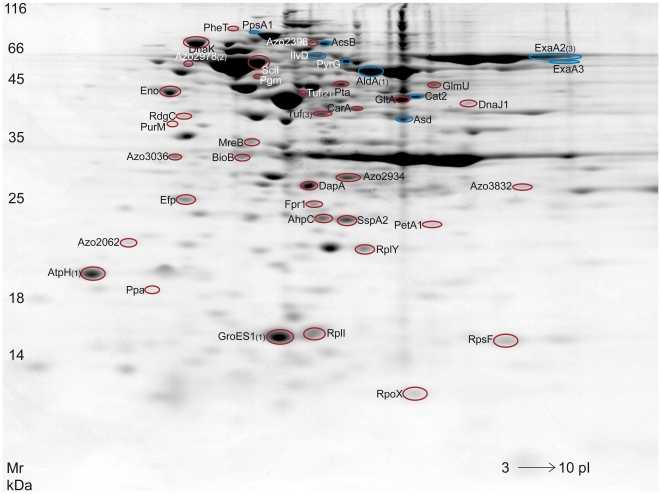
Protein pattern of *Azoarcus* sp. BH72 grown aerobically under standard conditions. Encircled spots have been identified by MALDI-TOF-MS. Proteins marked with blue circles showed increased intensity in cultures grown in conditioned supernatant, red circles indicate proteins with decreased levels.

To identify putative targets of density-dependent regulation also at protein level, the proteome of *Azoarcus* sp. BH72 grown in conditioned supernatant for four hours versus early exponential growth was compared. Two-dimensional gel electrophoresis was performed, and the protein patterns were compared with the Image master software package for 2D gel electrophoresis. Changes in protein spot intensity were determined from mean intensity values of four parallel gels each and only changes in intensity of at least 2.5 fold were taken into consideration. These experiments showed that 18.0% of all detected protein spots in the 2D-gels were differentially regulated; around 14.0% showed decreased intensities (49 protein spots) whereas 4.0% (12 protein spots) appeared to be present with higher intensities upon incubation in conditioned supernatant. Forty-four protein spots could be identified by mass spectrometry (Figure 2). Detailed protein parameters such as Gravy values, subcellular localization as well as occurrence of signal peptides and transmembrane domains and mass spectrometry data can be found in Table S4. In general, the proteomic approach supported the microarray data (see Table S3 and S4). Unfortunately, PilA could not be detected on the 2D protein gels, but with a theoretical pI of 9.7 and a mass of only 6403 Da PilA would not be expected in the analytical window employed [20].

The combination of transcriptomic and proteomic approaches revealed that exposure of exponentially growing *Azoarcus* sp. BH72 to conditioned culture supernatant had a strong impact on gene expression and protein synthesis. Altogether 440 genes or proteins of *Azoarcus* sp. BH72 were found to respond in expression or abundance, detailed results of all regulated genes and proteins are depicted in Figure S3. Several genes that were regulated in a cell density-dependent manner could be shown to display differences in the level of the corresponding proteins as well (Figure S3). For eight of the proteins that showed lower levels in the 2D-gels (encoded by *azo0086*, *azo0156*, *azo0718*, *azo0754*, *azo1062*, *azo1280*, *azo2396*, *azo3419*) in samples with conditioned supernatant, the mRNA levels were reduced as well. However, some discrepancies between the two approaches appeared: The genes *azo2062* and *azo3896* (only at 1 h) were found to be up-regulated in the transcriptome approach, whereas the corresponding proteins showed decreased levels in conditioned supernatants in the protein gels. Discrepancies might be caused by protein binding predicted for the protein domain FKBP_C in Azo2062, or to the timing of analysis (4 h for proteome).

### Quantitative PCR studies validated data obtained by microarray

Selected results obtained by the microarray approach were further examined by real-time RT-PCR. The expression of seven genes that showed differential expression in conditioned supernatant compared to exponentially growing cells was analyzed with 16S rRNA as it is often chosen as a reference [25], [26], [27], [28]. The data of this real-time PCR analyses (Table 1) corroborated the microarray results, that the genes *azo0156*, coding for the δ-subunit of ATPase, and *azo3412*, encoding the ribosomal protein L22P, were indeed down-regulated in conditioned supernatant with the factors −2.8±0.8 and −2.4±1.3, respectively. Moreover, the genes *azo0673* (11.5±4.6), *azo3674* (62.4±33.5), *azo3868* (44.9±6.2) as well as *azo3874* (40.2±2.5) were found to be up-regulated under the mentioned growth conditions. Only gene *azo3294* (1.0±0.1) could not be shown to be differentially expressed by real-time PCR studies, whereas its expression was highly up-regulated and well detectable in the microarray approach.

**Table 1:** Differential gene expression of *Azoarcus* sp. BH72 upon incubation in conditioned supernatant as detected by real-time PCR and microarray transcriptome studies.

Gene	Gene product	Induction	
		QPCRa	Microarray
*azo0156*	Putative ATP synthase, delta chain	−2.8±0.8	−3.5
*azo0673*	Periplasmic nitrate reductase accessory protein	11.5±4.6	4.6
*azo3294*	Protoheme IX farnesyltransferase	1.0±0.1	11.3
*azo3412*	50S ribosomal protein L22	−2.4±1.3	−2.5
*azo3674*	Serine protease	62.4±33.5	5.0
*azo3868*	Acetoin dehydrogenase, beta subunit	44.9±6.2	10.5
*azo3874*	Conserved hypothetical secreted protein	40.2±2.5	5.1

The induction factors obtained by microarray and real-time experiments showed some quantitative differences. This observation was not unexpected since a bias towards underestimating the magnitude of mRNA change has previously been described for oligonucleotide microarray data. Moreover, the fold changes of highly expressed genes and genes expressed at low levels are difficult to compare with the different methods applied [29], [30]. The direction of regulation mostly coincided between the microarray and quantitative RT-PCR approach. Accordingly, the microarray approach used here was suitable for monitoring gene expression changes in *Azoarcus* sp. BH72.

### Cellular processes such as type IV pili formation and regulation, nutrient transport and energy metabolism are affected by incubation in conditioned supernatant

**Figure 3:**
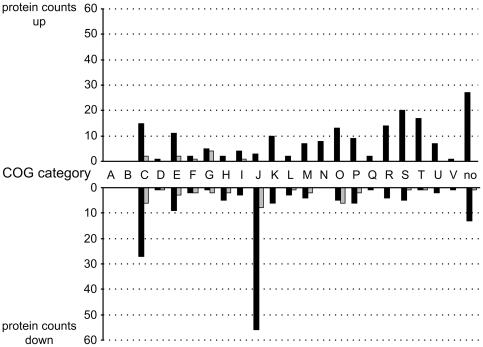
Distribution of differentially regulated proteins and genes of *Azoarcus* sp. BH72 according to COG categories. Differentially regulated proteins detected by two-dimensional gel electrophoresis are shown with grey bars and mRNAs identified by microarray with black bars. Activation in conditioned supernatant is indicated with “up” and repression with “down”. A: RNA processing and modification, B: Chromatin structure and dynamics, C: Energy production and conversion, D: Cell cycle control, mitosis and meiosis, E: Amino acid transport and metabolism, F: Nucleotide transport and metabolism, G: Carbohydrate transport and metabolism, H: Coenzyme transport and metabolism, I: Lipid transport and metabolism, J: Translation, K: Transcription, L: Replication, recombination and repair, M: Cell wall/membrane biogenesis, N: Cell motility, O: Posttranslational modification, protein turnover, chaperones, P: Inorganic ion transport and metabolism, Q: Secondary metabolites biosynthesis, transport and catabolism, R: General function prediction only, S: Function unknown, T: Signal transduction mechanisms, U: Intracellular trafficking and secretion, no: not in COG. Absolute protein counts are shown.

To gain better insights into the functions of genes and proteins differentially regulated, COG-categories according to Tatusov et al. [31] can be used for assignment of gene products (Figure 3). Several cellular processes were affected by incubation in conditioned supernatant in the grass endophyte. In general, energy production and conversion (C) as well as translation, ribosomal structure and biogenesis (J) were repressed. In contrast, genes for a variety of hypothetical proteins (R, S and no COG) and signal transduction mechanisms (T) appeared to be activated after incubation with conditioned culture supernatants. They are summarized in Table S5; particular processes are given in more detail below.

Many proteins that are involved in translation processes were differentially expressed, among them the bacterial translation initiation factor 3 (*infC*), elongation factors such as Efp, Tsf, TufA and TufB. Fourty-eight out of 53 genes coding for ribosomal proteins were found to be repressed according to the *Azoarcus* sp. BH72 microarray. Concordantly, the inhibitory influence of the conditioned culture supernatant was obvious for energy metabolic processes as for example all subunits of ATP synthase were down-regulated. Furthermore, almost all factors from the Nuo-cluster responsible for the formation of the NADH dehydrogenase complex in the respiratory chain were found to be repressed, and expression of genes for five electron transfer flavoproteins (EtfA1, EtfB1, EtfB2, EtfB3 and Etf1) were also found to be negatively affected. However, genes for specialized RNA-polymerase sigma factors, sigma-38 (RpoS) and sigma-24 (AlgU), were around 2.0-fold up-regulated; genes for regulators related to metal and iron uptake, such as the nickel responsive regulator (*nikR*), the phosphate regulon sensor proteins PhoR as well as the phosphate uptake regulator PhoU, and the ferric uptake regulator (*azo0644*) were around 2- to 3-fold up-regulated according to the microarray approach. Also other genes related to iron were differentially expressed: the genes for bacterioferritins Bfr1 and Bfr2 and the bacterioferritin-associated ferredoxin Bfd were activated, and three TonB-dependent receptor genes were differentially expressed (*azo2156*, *azo2396* and *azo3023*).

Related to the first known target *pilA*, eight genes localized in the *Azoarcus* sp. *pil*-clusters were found to be activated, encoding the putative type IV pili biogenesis proteins Azo1608, PilY1A and PilW, the prepilin like proteins Azo2180 and PilV, the type IV pilus assembly protein PilX, as well as the twitching motility protein PilU1. As expected, *pilA* was also detected among the regulated genes in the microarray with a fold expression of 2.9 after four hours incubation with conditioned culture supernatant.

Striking was the high amount (70) of genes affected in expression that encoded proteins belonging to the group of (conserved) hypothetical proteins or proteins that were poorly characterized. Some of those proteins were highly regulated, such as the conserved hypothetical membrane protein Azo2876 and the hypothetical secreted protein Azo0456 that showed 6.8-fold as well as 13.1-fold gene expression after four hours of incubation with conditioned culture supernatant, respectively. Moreover, the gene expression of *azo1684* was 10.9-fold up-regulated according to the microarray approach. The gene product shows a domain that is required for attachment to host cells in *Agrobacterium tumefaciens* and is therefore of high potential interest in the plant-associated bacterium *Azoarcus* sp. BH72.

Among 16 other (conserved) hypothetical secreted proteins whose gene expression was activated, Azo0275, Azo0347 and Azo3784 showed only poor sequence similarities to known bacterial proteins.

### The differential expression of four additional genes validated in response to conditioned supernatant

**Figure 4:**
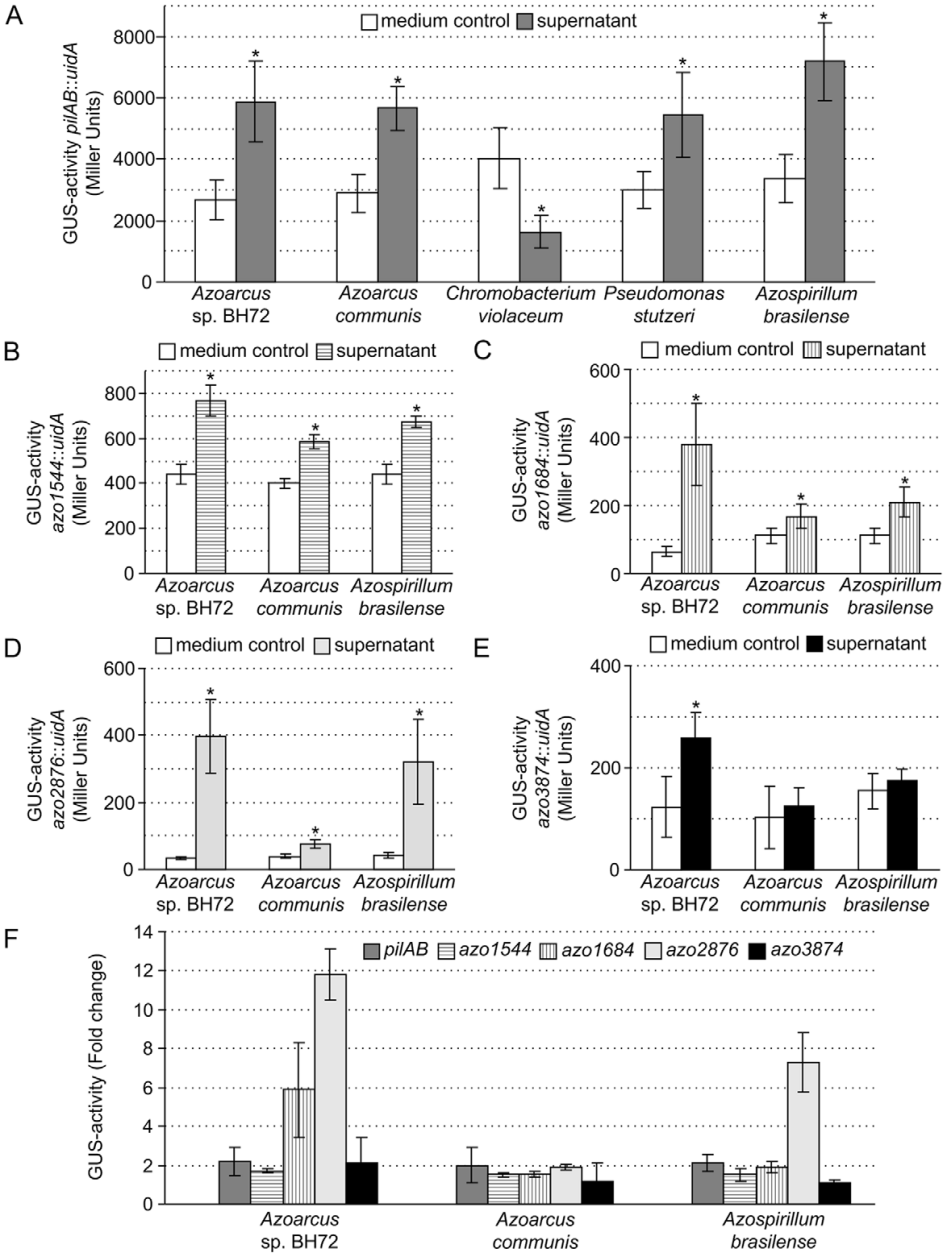
Expression of transcriptional gene fusions in conditioned supernatant. (A–F) Gene expression determined by ß-glucuronidase activities from respective transcriptional reporter gene fusions with *uidA*, in VM-ethanol medium. (A) Induction of *pilAB* gene expression in BHΔ*pilS*::pJBLP14 by supernatants of *Azoarcus* sp. BH72, *Azoarcus communis*, *Chromobacterium violaceum*, *Pseudomonas stutzeri* and *Azospirillum brasilense* obtained by supernatant bioassays after four hours of incubation. (B, C, D, E) Induction of *azo1544* (B), *azo1684* (C), *azo2876* (D) and *azo3874* (E) gene expression in the respective strains by supernatants of *Azoarcus* sp. BH72, *Azoarcus communis* and *Azospirillum brasilense* obtained by supernatant bioassays after four hours of incubation. (F) Fold changes of induction for all tested genes (*pilAB*, *azo1544*, *azo1684*, *azo2876* and *azo3874*) in comparison to each other. For all experiments, fresh medium was used instead of supernatant as negative control, and the values were set to one for calculation of fold changes. Standard deviation was calculated from at least three independent experiments. Stars indicate significance (at least *P<0.05*) as determined by unpaired t-test analyses.

The transcriptome profiling now led to the detection of new conditioned supernatant- regulated target genes, in addition to *pilAB*, allowing analysis of possible similarities of regulation patterns for different genes. Several up-regulated genes encoding proteins of different functions were selected. Up-regulated was the expression of the gene *azo3874*, encoding a conserved hypothetical secreted protein for which the Glo_EDI_BRP_like domain suggests functions related to glyoxylase I (catalyzing the glutathione-dependent inactivation of toxic methylglyoxal) or to antibiotic resistance proteins. Moreover, the genes *azo2876*, coding for a conserved hypothetical membrane protein, *azo1684*, encoding a conserved hypothetical protein, and *azo1544*, coding for a regulatory GGDEF/EAL/PAC/PAS domain-containing protein, were selected. For more detailed expression studies applying transcriptional reporter gene fusions, the plasmid integration mutants *Azoarcus* sp. strains BH*azo1544*, BH*azo1684*, BH*azo2876* as well as BH*azo3874* were constructed. These strains carry a transcriptional *gfp*::*uidA*-fusion to the target genes, in strains BH*azo1544* and BH*azo3874* the target genes are additionally inactivated. Upon incubation with conditioned supernatant from *Azoarcus* wild type, *azo1544*, *azo1684*, *azo2876* and *azo3874* gene expression was significantly induced after four hours compared to the negative control with VM-ethanol medium (Figure 4B, C, D, E and F).

The supernatant bioassays were routinely carried out with conditioned culture supernatants obtained from stationary cultures of *Azoarcus* sp. BH72 grown in VM-ethanol medium. However, this complex medium containing yeast extract and peptone might handicap analytical analyses for identification of the unknown signaling molecule(s). Therefore, growth of *Azoarcus* sp. BH72 was tested in the minimal medium SM-ethanol. In this medium the generation time of the wild type and the mutant BH*azo3874* was higher, however, cultures reached the stationary phase after overnight incubation at 37°C. The generation time of *Azoarcus* sp. wild type in VM-ethanol medium was 1.5±0.03 hours compared to 2.2±0.02 hours in minimal medium, whereas strain BH*azo3874* showed a generation time of 2.2±0.15 hours in SM-ethanol and 1.6±0.16 hours in complex medium. Bioassays with conditioned culture supernatants harvested from cultures grown in the two different media were performed with the reporter strains *Azoarcus* sp. BHΔ*pilS*::pJBLP14 and BH*azo3874* (data not shown). Expression of both genes was induced in presence of conditioned culture supernatant obtained from *Azoarcus* sp. cultures grown in complex VM-ethanol medium as well as grown in synthetic SM-ethanol medium.

### The signaling molecule(s) in conditioned supernatant is widespread among bacteria

The inducing ability of conditioned cell-free culture supernatants from different bacterial species and genera was tested using the *Azoarcus* sp. BHΔ*pilS*::pJBLP14 reporter strain system. The soil-borne species *Azoarcus evansii* did not produce active supernatant (data not shown), whereas supernatants of the Kallar grass-associated strain *Azoarcus communis* induced the *pilAB*::*uidA* gene expression 2.0-fold (5663±727 Miller Units, *P 0.0071*; Figure 4A). Other tested plant-associated or species of *Proteobacteria* did not lead to significant induction, such as *Azospirillum lipoferum*, the endophytes *Azonexus fungiphilus* and *Azovibrio restrictus*, and *Azotobacter vinelandii* (data not shown). The phytopathogenic bacterium *Chromobacterium violaceum* even led to significant inhibition of expression, presumably by antagonistic effects (Figure 4A), whereas supernatants from *P. syringae* and *X. oryzae* did not lead to a significant change in *pilAB* gene expression (data not shown). Surprisingly, the incubation with supernatants from *Pseudomonas stutzeri* strain DSM4166 isolated from the rhizosphere of *Sorghum mutan*s led to induction of the *Azoarcus* sp. *pilAB* gene expression (1.8-fold±0.62, 5451±1377 Miller Units, *P 0.0171*; Figure 4A). The incubation of the reporter strain *Azoarcus* sp. BHΔ*pilS*::pJBLP14 with supernatants from *Azospirillum brasilense* also enhanced the *pilAB* gene expression 2.1-fold (7194±1275 Miller Units, *P 0.0004*; Figure 4A). These observations indicate that a similar inducing molecule might be synthesized in several plant-associated bacteria from *Alpha*- and *Gammaproteobacteria*.

Gene induction by heterologous supernatant was also observed for the newly discovered target genes *azo1544*, *azo1684* and *azo2876*, although only slight induction could be shown for *azo1544*. The incubation of *Azoarcus* sp. strains BH*azo2876* with conditioned supernatants of *Azoarcus communis* or *Azospirillum brasilense* showed a fold change of 1.9 or 7.3, respectively (76±13 or 319±127 Miller Units, *P 0.0017* and *P 0.0199*; Figure 4D, F), whereas the expression of the gene was 11.8-fold (*P<0.0001*) increased in conditioned supernatants of strain BH72. The gene expression of *azo1684* was also significantly enhanced (Figure 4C, F) after incubation with supernatants from *Azoarcus* wild-type (5.9±2.45, 379±122 Miller Units, *P 0.0022*), *A. communis* (1.5±0.15, 168±36 Miller Units, *P 0.0376*) as well as *A. brasilense* (1.9±0.28, 319±127 Miller Units, *P 0.0075*).

### Gene *azo2876* up-regulated in conditioned supernatant is expressed in rice roots

**Figure 5:**
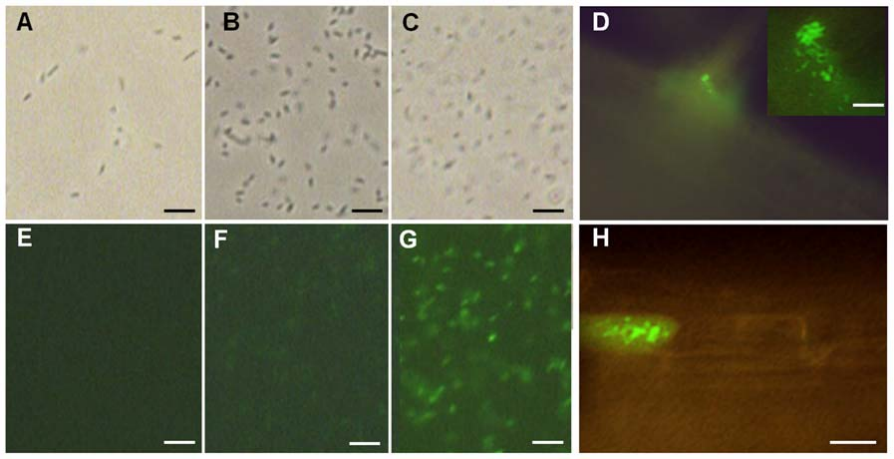
Expression of a transcriptional *azo2876::gfp* fusion in pure culture or during interaction with rice roots. Phase contrast (A–C) and corresponding fluorescence micrographs (E–G), of strain *Azoarcus* sp. BH*azo2876* expressing a transcriptional *2876::gfp* fusion in pure culture, or in infected rice roots (fluorescence micrographs D, H). Cells grown in VM-ethanol medium at exponential (A, E) or stationary growth phase (C, G), or in conditioned supernatant for 4 h (B, F); all fluorescence images taken with the same setting of the video camera. (D, H) Roots of rice seedlings 13 d after inoculation; bacterial GFP fluorescence at emergence points of lateral roots (D, with close-up in right corner, and in epidermal root cell (H). Bars correspond to 7 µm (A–C, E–G), 10 µm (D) and 20 µM (H).

As *pilA* is involved in root colonization, also some newly identified target genes that are up-regulated in conditioned supernatant might be expressed in association with the plant. We selected strain BH*azo2876* for a plant experiment, as this strain showed relatively high induction levels in GUS assays (Figure 4) which might allow differentiating induced from uninduced state by fluorescence. Single cell fluorescence can only be detected in *Azoarcus* sp. when *gfp* is expressed at relatively high levels [32]. Indeed weak (Figure 5 F) or strong fluorescence (Figure 5 G) could be detected in pure cultures only in conditioned supernatant or stationary growth phase, respectively, but not in exponentially growing cells (Figure 5 E). Strong bacterial fluorescence was visible at emergence points of lateral roots (Figure 5 D) and inside epidermal cells (Figure 5 H) of roots of rice seedlings inoculated with the reporter strain BH*azo2876*, indicating that this gene is highly expressed during infection of rice roots, as well.

## Discussion

Since type IV pili are one of the few known determinants for endophytic colonization by diazotrophic grass endophytes, knowledge on the transcriptional regulation of the pilus genes may give important insights into interaction strategies of endophytes with their hosts. Here we demonstrated that *pilAB* gene expression in *Azoarcus* sp. BH72 increases with population density and upon carbon starvation. Similar to quorum sensing-dependent mechanisms, expression was also elevated upon incubation in conditioned cell-free supernatant, albeit only in a *pilS* deletion mutant. In other bacteria such as *Pseudomonas aeruginosa*, the two component regulatory system PilR and PilS is required for the transcriptional regulation of *pilA* [33]. In strain BH72, PilR was identified as major transcriptional regulator for *pilAB* expression, although weak expression was not completely abolished in the *pilR* mutant [20]. A potential σ^54^-type promoter is localized upstream of *pilAB* of *Azoarcus* sp. strain BH72 [18], and results of primer extension analysis were in accordance with RpoN-dependent transcriptional activation by PilR. The *pilS* gene encodes a sensor kinase that is located at the pole of the cell [34], but the signal to which it responds is unknown [35]. Our results indicate that PilS directly or indirectly responds to the absence of carbon in the environment, leading to slightly enhanced *pilAB* expression. The inhibitory effect on density-regulated gene induction suggests that PilS might act as bifunctional sensor kinase acting negatively on expression when not phosphorylated, which is also supported by the effect of a point mutation in the putative phosphorylation site of PilS. In *Myxococcus xanthus* the *pilA* gene expression requires PilR, but not the sensor kinase PilS, which probably functions as a negative rather than a positive regulator [36].

According to our results, the molecule(s) that may confer the density-dependent regulation in *Azoarcus* sp. strain BH72 is likely to be a small hydrophilic molecule, and not AHL.

**Figure 6:**
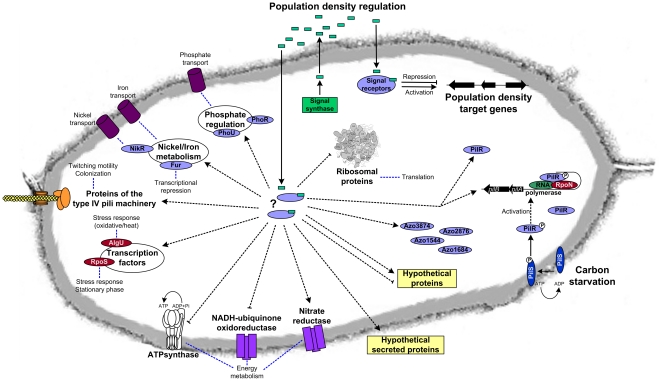
Schematic representation of regulation in *Azoarcus* sp. BH72 in conditioned supernatant. Summary of major processes affected in conditioned supernatant in comparison to medium control. Activation of gene expression is indicated by dotted black arrows whereas repression is shown with blocked dotted lines. Direct activation and repression may lead in turn to further regulation processes, e.g. through regulatory proteins such as Fur, PilR or transcription factors; thus blue dotted lines indicate processes that might be indirectly affected.

Furthermore, a combination of proteomic studies based on two-dimensional gel electrophoresis and MALDI-TOF-MS with expression profiling by whole genome microarray was used to characterize additional genes that are affected in their expression in presence of conditioned supernatant in *Azoarcus* sp. strain BH72. Many target genes were detected which were differentially regulated, suggesting global regulatory mechanisms. Likely, not all genes are under an autoinducer-mediated control, as conditioned supernatants also contain secreted metabolites that may interfere with QS-dependent regulation processes. The comparative studies revealed that 18% of the detected proteins and 8% of the transcriptome were differentially regulated triggered by addition of conditioned supernatant, which was supplemented with carbon source (ethanol) to overcome putative starvation effects. In Figure 6 the major regulated processes in *Azoarcus* sp. BH72 are summarized. Thus, this study provided the basis to study the putative cell density-related regulon(s) in strain BH72 in more detail.

For the genes *azo1544*, *azo1684*, *azo2876* and *azo3874* gene expression was newly discovered to be population density-dependent in *Azoarcus* sp. BH72. They were selected for further comparative transcriptional studies by reporter gene fusion, as they are localized in a different genomic region than *pilA* (*azo3355*), and the proteins are not obviously functionally related to the type IV pilus machinery. Our analysis showed many similarities in gene expression patterns suggesting similar regulatory circuits, but also some differences to *pilA* regulation. For example, *azo3874* expression was already induced 1.9- (GUS-activity) to 5.1-fold (microarray) after incubation with conditioned culture supernatant for just one hour, representing a relatively fast and robust reporter system. Similar response patterns were also detected with respect to inducer production in different culture media and by different bacterial species: The *pilAB* as well as the *azo3874* gene expression were induced by conditioned culture supernatants obtained from *Azoarcus* wild type cultures grown in the minimal medium SM-ethanol, thus the putative signaling molecule(s) can be synthesized in minimal medium and not only in complex medium containing peptones and yeast extract. This will facilitate elucidation of the chemical structure of the inducing molecule(s). *PilA* expression was induced in conditioned supernatant not only from *Azoarcus* sp. BH72, but also from some other albeit not all tested bacterial species and genera. This also extended to other putative density-regulated target genes, *azo1684* and *azo2876*, whose expression was induced by supernatants produced by the Kallar grass root isolate *Azoarcus communis* and the root-associated alphaproteobacterium *Azospirillum brasilense*. This suggests that the putative signaling molecule(s) or metabolite is more widespread and might be used for interspecies communication.

In contrast to the *pilAB* gene expression studies that were carried out with the reporter strain *Azoarcus* sp. BHΔ*pilS*::pJBLP14 in the *pilS* deletion background, experiments with *Azoarcus* sp. BH*azo1544*, BH*azo1684*, BH*azo2876 and* BH*azo3874* were carried out in the wild type background. Induction of the mentioned genes was thus induced by conditioned culture supernatant in the presence of the histidine kinase PilS. This demonstrates that the putative signal cascade in *Azoarcus* sp. does not require *pilS* deletion for gene induction in our reporter gene assays, which is also corroborated by high induction values for many genes in microarray and RT-PCR experiments in the wild type background.

It has already been shown that the response regulator PilR is required as a transcriptional activator for the *pilAB* gene expression of *Azoarcus* sp. BH72 and plays therefore a role in type IV pili biogenesis [20]. In general, pili are involved in plant-microbe interactions and they are responsible for twitching motility and adhesion to eukaryotic cells and therefore important for colonization processes [18], [19]. It could be shown that eight proteins involved in type IV pili formation, assembly or twitching motility and the response regulator PilR itself were under positive control in conditioned medium. These observations connect the regulation mechanisms to broader type IV pili- mediated processes than just the structural pilin gene expression. In *Pseudomonas aeruginosa* it was shown that type IV pili assembly and function is dependent on the three interrelated QS systems [37], [38], [39]. The comparison of PilR-regulated proteins [20] with the newly identified cell density-dependent targets revealed an overlap of 35 proteins or genes that were differentially regulated (Table S3). This suggests that PilR may be part of the putative signaling cascade in *Azoarcus* sp. BH72, albeit it is not likely to be the primary sensor for a signaling molecule, since many genes or proteins differentially regulated in conditioned supernatant were not differentially synthesized in the PilR mutant (Table S3).

As many regulatory proteins, several posttranslational modification proteins and transcription factors were regulated in conditioned supernatant, the regulatory cascade appeared to involve a complex stimulon. In the future such regulatory proteins need to be studied in more detail to investigate their position in the regulatory hierarchy. The RNA polymerase sigma factors RpoS (sigma-38) and AlgU (sigma-24) were also found to be regulated upon incubation of *Azoarcus* sp. BH72 in conditioned supernatant and may thus contribute to indirect effects of the cascade. In general, sigma factors act as transcription initiation factors and they can be activated in response to different environmental stimuli [40]. For *Pseudomonas aeruginosa* it was stated that the alternative sigma factor RpoS represses *rhlI* gene expression and therefore plays a role in QS [41].

Special proteins are required for detection of signaling molecules and subsequent signal transduction processes. It is likely that the expression of genes coding for such proteins is activated in conditioned supernatants as well. Nine candidate genes, encoding probable regulatory proteins were discovered with the microarray approach: *azo0622*, *azo0898*, *azo1544*, *azo2073*, *azo2408*, *azo2672*, *azo3330*, *azo3436* and *azo3498*. In the future, the involvement of such proteins in signal transduction needs to be studied in more detail to investigate their position in the regulatory hierarchy.

 Several proteins that are involved in iron metabolism and iron storage (Bfr1, Bfr2 and Bfd) were positively regulated in conditioned supernatant. Also expression of the ferric uptake regulator Fur (*azo0644*) was activated in *Azoarcus* sp. BH72. Typically, Fur acts as a transcriptional repressor by binding to regulatory Fur box sequences in the promoters of iron-regulated genes under iron-repleted conditions. This protein also acts as a global regulator controlling the expression of iron acquisition and storage genes as well as the expression of genes involved in the oxidative stress response, virulence genes and small, iron-repressible regulatory RNAs [42], [43], [44]. The activation of the gene *azo0644*, encoding the Fur protein, in conditioned supernatant would repress the expression of Fur-dependent genes in *Azoarcus* sp. BH72, and this process would in turn lead to indirect regulation processes integrated in a complex regulon. Also nickel is an essential trace element for prokaryotes as it forms the active centre of metalloenzymes. When present at high concentrations nickel inhibits growth and exhibits a toxic effect. Therefore, the expression of the nickel-specific transport system is under tight control of the metallo-regulatory protein NikR that specifically responds to nickel [45]. In the presented study NikR was shown to be up-regulated. Thus, metal uptake and storage appear to be controlled in a cell density-dependent manner in *Azoarcus* sp. BH72.

In total 70 (conserved) hypothetical proteins or proteins with unknown functions were found to be differentially regulated in conditioned supernatant. Their differential expression (23% of all detected genes or proteins) was strongly overrepresented in comparison to their abundance in the genome (15%). This indicates that particularly as yet unknown cellular functions are controlled in a density-dependent manner. As an example, gene *azo2876* encoding a hypothetical membrane protein was validated to be induced in conditioned supernatant based on transcriptional reporter gene assays. Interestingly, strong promoter activity of this gene was also detected in bacteria colonizing rice roots, suggesting that some of the new density-regulated target genes in *Azoarcus* might also play a role during root colonization.

 This study is the first global approach to initiate characterization of a novel quorum sensing system in the model grass endophyte *Azoarcus* sp. BH72. Comparison of the transcriptome and proteome data from conditioned supernatants of *Azoarcus* sp. BH72 with the QS regulon of other bacteria revealed, that several similar sets of genes and proteins were controlled [39], [46], [47], [48], [49]. As these studies were based on different autoinducers, such as *N*-acyl-homoserine lactones or Autoinducer-2, regulation of target genes may be overlapping even when different “languages” are used by bacteria.

## Materials and Methods

### Bacterial strains and growth conditions

In general, *Azoarcus* sp. strains (Table S1) were grown aerobically under standard growth conditions in VM-ethanol medium (per L 0.4 g KH_2_PO_4_, 0.6 g K_2_HPO_4_, 1.1 g NaCl, 0.5 g NH_4_Cl, 0.2 g MgSO_4_, 26 mg CaCl_2_, 10 mg MnSO_4_, 2 mg Na_2_MoO_4_, 66 mg Fe(III)-EDTA, 1 g yeast extract, 3 g bacto peptone, 6 mL ethanol, pH 6.8) at 37°C with constant shaking at 180–200 rpm. For bacterial cultivation the Erlenmeyer flasks were only filled up to 1/10 of the total volume to assure sufficient aeration.

For production of conditioned supernatants the following strains were used: *Azoarcus evansii* KB740, *Azospirillum lipoferum* Sp59b, *Azospirillum brasilense* Sp7, *Azoarcus communis* SWub3, *Chromobacterium violaceum* ATCC31532 (30°C), *Xanthomonas oryzae* PXO99 (30°C), *Azotobacter vinelandii* MV531 (30°C), *Pseudomonas syringae* DC3000 (30°C), *Azonexus fungiphilus* BS5-8, *Azovibrio restrictus* S5b2 and *Pseudomonas stutzeri* DSM4166 (30°C), which were all grown on VM-ethanol medium at 37°C if not mentioned otherwise.

For AHL detection assays *Escherichia coli* and *Chromobacterium violaceum* were grown aerobically on Luria-Bertani-medium (LB) at 37°C or 30°C [50], and *Pseudomonas putida* on modified LB medium containing 4 g NaCl per L at 30°C [51]. *Rhizobium* sp. NGR234 was grown on TY medium [52] at 30°C. Antibiotics used for *E. coli* or *Azoarcus* strains were tetracycline (12.5 µg per mL), chloramphenicol (12.5 µg per mL), ampicillin (150 or 30 µg per mL), kanamycin (30 µg per mL or for *C. violaceum* 25 µg per mL). Gentamicin (25 µg per mL) was used in the cultivation of *Pseudomonas putida*.

For the proteomic approach pre-cultures of *Azoarcus* sp. BH72 were grown in 100 mL VM-ethanol medium until the early exponential growth phase (OD_578 nm_ of 0.3). For growth under “quorum sensing”-like conditions, these cultures were incubated for four hours with four volumes (400 mL) of conditioned culture supernatant from *Azoarcus* sp. BH72 wild type in a 5 L Erlenmeyer flask. As controls, *Azoarcus* sp. BH72 was grown in 500 mL VM-ethanol medium until the exponential growth phase. Cells from three independent cultures were harvested by centrifugation (4°C, 15 min, 6,300 g), pellets were resuspended in cold PBS (14 mM NaCl, 0.27 mM KCl, 1.65 mM Na_2_HPO_4_, 0.15 mM KH_2_PO_4_, pH 7.0), centrifuged again and stored at −80°C until protein extraction. For collection of conditioned culture supernatants, containing the unknown signaling molecule, strain BH72 was grown in VM-ethanol medium at 37°C. After 24 h pre-cultures were diluted 1∶6 with fresh medium and incubated for another 24 h. The optical density at 578 nm was measured and only supernatants from cultures that reached the stationary growth phase (OD_578_>1) were harvested by centrifugation with 12,857 g for 20 min at room temperature, the supernatants were supplemented with 3 mL ethanol per L and subjected to supernatant bioassays without filtration.

For the comparative transcriptomic study, *Azoarcus* sp. BH72 was grown until the early exponential growth phase and under “quorum-sensing”-like conditions (see above) for one or four hours, respectively. In three biological independent replicates for each condition tested, the cells from two cultures were harvested quickly by centrifugation (room temperature, 5 min, 6800 g), pellets were suspended in PBS, pooled, centrifuged again and stored at −80°C until RNA isolation.

### Construction of mutants and reporter strains

The plasmids used in this study are listed in Table S1. They were constructed as follows: The transcriptional fusions of reporter genes with *pilAB* were generated by cloning an 1.8 kb *uidA* gene, coding for the reporter enzyme β-glucuronidase, or 0.7 kb *gfp* gene, encoding the green fluorescent protein, into the *Mfe*I site of the plasmid pJBLP1, resulting in pJBLP14 (carrying *pilAB*::*uidA*) [20] or pJBLP1*gfp* (carrying *pilAB*::*gfp*).

Plasmid integration mutagenesis was used for inactivation of the genes *azo0390*, *azo1746*, *azo3178* and *azo3379*. Truncated gene fragments (500–600 bp) starting close to the 5′ end but lacking the start codon and carrying a stop codon instead, were amplified by PCR. PCR products were cloned into the pPCR-Script™ AmpSK(+) vector and subcloned into the conjugative vector pK18*mobsacB* with *Hin*dIII-*Xba*I. The final constructs were conjugated into *Azoarcus* by triparental mating, and the correct integrations were confirmed by Southern blot analysis.

To study the gene expression of *azo1544*, *azo1684*, *azo2876* and *azo3874*, a transcriptional reporter gene fusion with *uidA* encoding the ß-glucuronidase and in tandem *gfp* encoding green fluorescent protein was generated. Insertional mutants were constructed by integrating plasmids pK18GGST-1544, pK18GGST-1684pro, pK18GGST-2876pro and pK18GGST*azo3874*, carrying a fusion of the promoterless reporter genes *gfp* and *uidA* to the respective *Azoarcus* gene, by homologous recombination into the *Azoarcus* sp. BH72 chromosome. This cloning strategy also inactivated genes *azo1544* and *azo3874*.

For deletion of the *pilS* gene, a 0.9 kb *Bsa*BI-*Nru*I fragment of pJBLP231 was excised, yielding an in-frame deletion of *pilS* (pJBLP2311) consisting of amino acid 107–407. The 1.8 kb *Sma*I fragment of pJBLP23 was exchanged by the mutagenized 0.9 kb *Sma*I fragment of pJBLP2311, yielding pJBLP232. The 3.2 kb insert of pJBLP232 was subcloned into the *Eco*RI-*Hin*dIII restriction site of pK18*mobsacB* for sucrose selection [53]. The final construct pJBLP234 was conjugated into *Azoarcus* sp. by triparental mating. Transconjugants selected for single recombination with kanamycin were further selected on 6% sucrose media for plasmid deletion. PCR and Southern blot analysis confirmed the chromosomal deletion of *pilS*. For point mutation of the conserved histidine residue putatively required for phosphorylation of PilS, in a short 0.6 kb *Pst*I-fragment of *pilS* (pUC-SC27) this histidine codon was exchanged to an arginine codon (CGC) (pUC-SC27M), and the resulting fragment enlarged by successive addition of 5′- and 3′- fragments of *pilS* (pUC-SC27M+3′+5′). To allow sucrose selection, the mutagenized *Eco*RI-*Hin*dIII fragment was subcloned into pK18*mobSacB*, and a double recombinant was selected after transfer into strain BH72 (BH*pilS*M). For reporter gene studies, the *pilAB*::*uidA* fusion was integrated into the chromosome (BH*pilS*M::pJBLP14).

### Supernatant bioassays and determination of β-glucuronidase activity

The impact of conditioned culture supernatants from *Azoarcus* sp. and other species (see above) on the gene expression of *pilAB*, *azo1544*, *azo1684*, *azo2876* and *azo3874* were monitored by reporter protein (β-glucuronidase) studies using a transcriptional *pilAB*::*uidA* (*Azoarcus* sp. BHΔ*pilS*::pJBLP14), *azo1544*::*uidA* (*A.* sp BH*azo1544*), *azo1684*::*uidA* (*A.* sp. BH*azo1684*), *azo2876*::*uidA* (*A.* sp. BH*azo2876*), or *azo3874::uidA* (*A.* sp. BH*azo3874*) fusion, respectively. These reporter strains (Table S1) were routinely grown in VM-ethanol medium at 37°C. When the cells had reached the early exponential growth phase, they were diluted with four volumes of conditioned culture supernatant and afterwards further incubated for one, two, three or four hours, respectively. As negative controls, cultures were incubated with fresh medium instead of supernatant. The conditioned culture supernatants were either harvested from wild type cultures grown in complex medium (VM-ethanol, see above) or minimal medium (SM-ethanol, consisting of modified VM-ethanol medium containing (per L) only 0.1 g NaCl, 0.1 g yeast extract, and no Bacto peptone. The production of conditioned culture supernatant was performed in 250 or 300 ml Erlenmeyer flasks with a total volume of 30 mL. The subsequent supernatant bioassays were carried out in 100 mL Erlenmeyer flasks with a final volume of 10 mL.

β-Glucuronidase activity was assayed according to [54] with modifications, in a buffer containing 60 mM Na_2_HPO_4_ and 40 mM NaH_2_PO_4_ (pH 7), 1 mM EDTA, 14 mM β-mercaptoethanol, 0.05% SDS and 2 mM p-nitrophenyl β-D-glucuronide. Reactions in 0.65 mL volumes at 37°C were stopped by adding 0.2 mL 2.5 M 2-amino-2-methylpropandiol, and the absorbance of p-nitrophenol was measured at 420 nm. β-Glucuronidase activity was calculated in Miller Units defined by: (A_420 nm_×1000)/(t (min)×OD_600 nm_). The GraphPad InStat software package (GraphPad software, San Diego, CA) was used for statistical analysis carried out by unpaired or paired T-tests, respectively.

### AHL detection assays based on sensor strains and AHL extraction

For cross-streak experiments, the test strain *Azoarcus* sp. BH72 or *Rhizobium* sp. NGR234 as a positive control were streaked close to the GFP(ASV)-based AHL sensor strains *E. coli* JM105 (pJBA89) or *P. putida* IsoF (pKR-C12) on VM-ethanol plates to form a T. After 24 h of incubation at 30 or 37°C, the green fluorescence of the AHL sensor strains were visualized by excitation with blue light, and the results were documented with a Hamamatsu Color Chilled 3CCD camera mounted on a binocular (Olympus SZX12). For the AHL plate detection assay, single colonies of AHL monitor strains *P. putida* IsoF (pKR-C12) or *E. coli* JM105 (pJBA89) were resuspended in 0.9% sodium chloride and plated onto LB agar plates. If *C. violaceum* CV026 was used, 100 µL of the reporter culture was mixed with 3 mL of LB soft agar and poured on LB agar plates. The test samples were filled up in holes punched into the agar. The plates were incubated at 30/37°C for 24 h. For analysis either the GFP fluorescence was quantified in a Fluoroimager Typhoon 8600 (GE Healthcare, Freiburg, Germany), or by visual inspection for violacein production. Putative AHL molecules were extracted with one volume dichlormethane from culture supernatants (grown in VM-ethanol medium to stationary phase). The extracts were evaporated at 40°C to dryness. Residues were dissolved in 1 mL acetonitrile following volume reduction to 0.1 mL by rotational evaporation. The liquid phase and the extracts were then applied to the AHL plate detection assay and/or to the quorum sensing bioassay (described above). For size fractionation, conditioned supernatant was first passed through an ultrafiltration membrane YM10, then through YM1 (Amicon, Bedford, USA).

### Infection of *Oryza sativa* seedlings with *Azoarcus* sp. BH*azo2876*

Rice seedlings (*Oryza sativa ssp. japonica cv. Nipponbare*) were surface-sterilized, germinated and inoculated with *Azoarcus* sp. BH*azo2876* as described [15], [55] with the following modifications: Germination occurred for 3 days at 30°C in the dark, followed by 2 days at 30°C, 15 kLux light intensity, 80% humidity and 14 /10 hours day/night intervals. Plant medium [55] contained 20 mg neutralized DL-malic acid per L, and seedlings were incubated at 30°C, 15 kLux light intensity, 80% humidity and 14/10 hours day/night intervals for 13 days.

### Fluorescence microscopic analysis of infected rice roots

13 days after inoculation roots were washed in 0.9% NaCl solution to remove remaining sand, separated from the shoot and rice grain. Roots were incubated for 30 min at 4°C in phosphate buffered saline to allow oxidation of GFP and placed on glass slides in 10% phosphate buffered saline, 90% glycerol and 0.25% of the antioxidant 1,4-Diazabicyclo(2,2,2)octan (DABCO) (pH 8.6 adjusted with HCl/ NaOH). For microscopic analysis an Axioplan 2 fluorescence microscope from Zeiss (Jena, Germany), equipped with a C5180 camera from Hamamatsu Photonics K.K., (Hamamatsu, Japan) was used [56].

### PAGE and Western blot analysis

Sodium dodecyl sulfate (SDS)-soluble cellular proteins were extracted [15] from equal amounts of cells from liquid cultures, and subjected to Tris-Tricine polyacrylamide gel electrophoresis (PAGE) followed by Western blot analysis with anti-PilA rabbit polyclonal antibodies (1∶5000) [18].

### Two-dimensional gel electrophoresis

Protein extraction, two-dimensional gel electrophoresis, and gel analysis was performed as described in [20] with 500 µg of protein extracted from *Azoarcus* sp. BH72. Only those proteins that showed at least 2.5-fold change in level between the two tested conditions were considered to be affected by conditioned culture supernatant.

### Protein identification by MALDI-TOF-MS

Identification of proteins by MALDI-TOF-MS/MS was performed as described in Hauberg *et al.* 2010 [20].

### RNA isolation and primer extension

For the “hot phenol” extraction method [57], bacterial cells from a 50 ml culture were resuspended in a 1∶1 mixture of prewarmed phenol-chloroform (pH 4.7) and 50 mM sodium acetate/10 mM EDTA/1% SDS (pH 5.1). Cells were incubated for 5 min at 65°C followed by 10 min incubation on ice. For phase separation the mixture was centrifuged for 10 min at 12°C and 8000 g, and the upper phase was mixed with the same volume of phenol-chloroform (pH 4.7). This phenol extraction was repeated three times followed by extraction with chloroform-isoamylalcohol (24∶1). RNA precipitation was performed with the same volume of isopropanol for 45 min on ice. The RNA was pelleted by centrifugation (12900× g, 10 min, 4°C) and washed with 70% ethanol. After drying RNA was dissolved in 1× RNAsecure (Applied Biosystems), and contaminating DNA was removed from RNA preparations by using the RNeasy Mini Kit (Qiagen) according to manufacturer's instructions. Finally, RNA was frozen in liquid nitrogen and kept at −80°C until further processing. Contaminating DNA was removed from RNA preparations by DNase I using Qiagen columns (RNeasy Mini Kit, Qiagen, Hilden, Germany) according to manufacturer's instructions.

Primer extension analysis was performed by standard procedure [58]. The primer (5′-GCAGTTTCTTCATTTCAATTCTCC-3′) annealed in *pilA*, 42 bp downstream of the predicted transcriptional start. It was end-labeled by ^35^S-α-ATP, mixed with 30 µg RNA, and extended using avian-myleoblastosis-virus reverse transcriptase (Roche, Mannheim, Germany). The cDNA was purified via extraction with 50% phenol, 49% chloroform and 1% isoamyl alcohol followed by ethanol precipitation. A DNA sequencing reaction [59] with the same oligonucleotide primer was run on the denaturing gel next to the cDNA and evaluated by autoradiography.

### Microarray processing and data analyses

20 µg of total RNA from one biological sample was reverse transcribed with BioScript RT in respective reaction buffer (Bioline) with amino modified random hexamers for 90 min at 42°C. For two-color labeling, the aminoallyl-labeled first strand cDNA from the two different RNA samples was coupled to fluorescent Cy3-NHS or Cy5-NHS esters (GE Healthcare), respectively. DNA contamination was excluded by PCR, and the quality of fluorescently labeled cDNA was checked on an agarose gel and with a Typhoon scanner (GE Healthcare, Fairfield, CT; USA) at 800 pmt, medium sensitivity and with the respective filters for Cy3 (green 532 nm laser, 555 BP20) and Cy5 (red 633 nm laser, 670 BP30) detection.

For construction of the oligonucleotide microarray, a collection of gene-specific 70mer oligonucleotide probes (designed and synthesized by Eurofins MWG Operon) for the 3,992 predicted protein-coding genes from *Azoarcus* sp. BH72 was spotted on epoxysilane-coated Nexterion Slide E (Schott) (CeBiTec, University Bielefeld). The array consisted of 17,280 single spots, spotted in rows of 20×18 per grid, which were arranged in 4×12 grids. Each gene was spotted in quadruplicates; five positive controls were spotted in additional replicates (*azo1072* (30S ribosomal protein S1), *azo1081* (50S ribosomal protein L35), *azo2104* (30S ribosomal protein S15), *azo2837* (probable glyceraldehyde 3-phosphate dehydrogenase) and *azo2898* (30S ribosomal protein S16). Negative buffer controls, as well as negative controls consisting of a rice-gene targeted oligonucleotide (Os05g0438800 similar to *actin1*) and randomized negative control oligonucleotides (human H2NC00001, H2NC00002, H2NC00003, H2NC00004, H2NC00005, H2NC00006; mouse M2NC000001, M2NC000005, M2NC000006, M2NC000008, M2NC000009, M2NC000010, M2NC000012; customized by Eurofins) which do not match to the genome of *Azoarcus* BH72 were also included. Prior to hybridization, samples were denatured at 65°C for 8 min. Hybridization was performed at 42°C for 16 h in Easy hybridization solution (Roche) supplemented with 5 µg salmon sperm DNA in a final volume of 60 µL under a cover slip in a hybridization chamber in a water bath. Afterwards the slides were washed once in 2× SSC/0.2% SDS for 5 min at 42°C, twice in 0.2× SSC/0.1% SDS for 1 min each at room temperature, twice in 0.2× SSC for 1 min each at room temperature and for 1 min on 0.05× SSC at 21°C. The glass slides were dried by centrifugation (7 min, 650 g, room temperature), and the Cy3- and Cy5-fluorescence was scanned with the GenePix™ Scanner 4000A (Molecular Devices) with a pixel size of 10 µm.

Image analysis was performed with the GenePix 4.1 program, and subsequent analyses were carried out with the open-source software TM4 [60] (http://www.tm4.org/). Before intensity values that were measured with GenePix could be compared, LOWESS normalization with MIDAS v2.19 was performed. The normalized data for each spot were aligned to the corresponding *Azoarcus* sp. strain BH72 gene name. Three independent experiments including dye-swap were performed and average expression folds were obtained from the replicates. A one-tailed paired t-test was performed with Bonferroni correction, and only genes that showed an expression of at least 1.8 fold and a P-value≤0.05 were regarded as being differentially expressed.

The microarray data are MIAME compliant and have been deposited in the GEO database (http://www.ncbi.nlm.nih.gov/geo) under accession no. GSE25939, GPL11300.

### Real-time PCR

Synthesis of cDNA was achieved by using gene specific reverse primers for genes *azo0156*, *azo0673*, *azo3294*, *azo3412*, *azo3674*, *azo3868*, *azo3874* and 16S rRNA with the Verso 2-Step QRT-PCR Kit from ABgene. 30 ng of total RNA was reverse transcribed in a total volume of 20 µL for 30 min at 42°C, followed by a denaturation step for 2 min at 95°C. 0.5 µL to 1.5 µL of the cDNA were used for the quantitative PCR step with 1×2-Step QPCR Mix (ABgene) and 0.5× SYBR green I dye (Molecular Probes) in a total volume of 25 µL. The QPCR was carried out in the Chromo 4 PTC-200 real-time PCR cycler (MJ Research) with the following program: Initial denaturation and enzyme activation for 15 min at 95°C linked to a loop of 40 cycles each having a denaturation step for 15 sec at 95°C, 30 sec at 60°C for annealing followed by an elongation step for 30 sec at 72°C. The PCR was followed by a melting curve from 60°C to 99°C with 0.5°C steps for 10 sec. The 2^−ΔΔ*C*^_T_ method was applied for data analyses, and 16SrRNA was used as a reference with the following primers: 16SRTfor: CTTGACATGCCTGGAACCTT, 16SRTrev: ATGACGT GTGAAGCCCTACC, azo0156for: ATCAACGATCCCAAGCTTTC, azo0156rev: CG TGTTCGTTCTTCAGAGCA, azo0673for: TCAGGAGGTGGGCAACTG, azo0673rev: ACAAGAACCGCCGTCCAC, azo3294for: CACGCAAAGATGATCAGGAA, azo3294rev:TGATCTACACCCTGCTGCTG, azo3412for: GAAACGCTTGAGGGTA GTGC, azo3412rev: GCTGAACATTCTGGCCTTCT, azo3674for: AGTTCAAGGCC AAGGTGCT, azo3674rev: CGTAACGGAGTTTTCGAAGC, azo3868for: CACTCGC AGTGCCTGTACTC, azo3868rev: CCCTCGAAGTAGGACATCCA, azo3874for: CCTTCAAGTTCGAGGACGAC, azo3874rev: ACGTAGAAGGCCAGGTGATG.

## Supporting Information

Table S1 **Strains and plasmids used in this study.**
(PDF)

Table S2 **Characterization of the autoinducer molecule in conditioned supernatant, determined as** ***pilAB*** **::** ***uidA*** **-inducing activity in supernatant bioassays with strain** ***Azoarcus*** **sp. BHΔ** ***pilS*** **::pJBLP14a.**
(PDF)

Table S3 **Differentially expressed genes detected by microarray analyses in** ***Azoarcus*** **sp. BH72 upon incubation in conditioned supernatant.**
(PDF)

Table S4 **Detailed parameters and mass spectrometry data for proteins in** ***Azoarcus*** **sp. BH72 differentially synthesized upon incubation in conditioned supernatant, as discovered by MALDI-TOF-MS.**
(PDF)

Table S5 **List of differentially regulated proteins and genes of** ***Azoarcus*** **sp. BH72 and their corresponding COG category.**
(PDF)

Figure S1 **Analysis of pilAB expression.** (A) Representation of the *pilAB* region in the chromosome of *Azoarcus* sp. strain BH72 and primer extension analysis. The DNA sequence upstream of the *pilAB* operon is labeled for a σ^54^-dependent promoter site (−24/−12, bold italics), the transcriptional start point (+1, small box) and the ribosome binding site (RBS, underlined). Below, primer extension analysis of the start site of the *pilAB* transcript. Lanes G A T C contain mixtures from DNA sequencing reactions performed with the primer complementary to the sequence of *pilA* 42 bp downstream of the predicted transcription start. In lane 1 the primer extension reaction from RNA extract of wild type cells was loaded, and the longest transcript labeled with a star. (B) Expression of a chromosomal *pilAB*::*uidA* fusion in wild type and Δ*pilS* mutant background under conditions of carbon starvation. Cells were grown in a pre-culture of complex medium (VM-Ethanol), washed two times with synthetic medium (SM) with or without the carbon source potassium malate (medium replacement), and then grown for 1 hour in the respective medium with or without carbon source (+/− C). Error bars indicate standard deviations from three replicates. Similar results were obtained in at least three independent experiments. Statistical analysis: columns labeled with different letters differ statistically significantly from each other (*P*<0.0001, unpaired t-test).
(TIF)

Figure S2 **Differentially regulated gene clusters as identified by transcriptome microarray upon incubation in conditioned supernatant.** (A–D), Four representative gene clusters of *Azoarcus* sp. BH72 are shown. Activated clusters are depicted with arrows showing upwards, and repressed clusters with arrows showing downwards, detected at the distinct time point of one or four hours of incubation, respectively. Stars indicate genes whose expression in the cluster was only changed after four hour (grey stars) or after one and four hours (black stars) of incubation with conditioned culture supernatant. Bold grey arrows refer to the direction of transcription with the respective gene name in black letters. Locations in the *Azoarcus* genome are depicted with grey numbers.
(TIF)

Figure S3 **Comparison of differential regulation in** ***Azoarcus*** **sp. BH72 upon incubation in conditioned supernatant, revealed by transcriptomic (T) and proteomic (P) approaches.** Colours in heat map indicate the fold-change in gene expression or protein synthesis, respectively, in conditioned supernatants. Colour code: light green = ≥+1.8, dark green ≥+2.0 for transcriptomic study and ≥+2.5 for proteomic study, orange ≤−1.8, red ≤−2.0 for transcriptomic study and ≤−2.5 for proteomic study, yellow = no change, white = not detected in 2D-gels).
(TIF)
