# Supplementary material for: Environmental Factors Affecting the Expression of pilAB as Well as the Proteome and Transcriptome of the Grass Endophyte Azoarcus sp. Strain BH72
Source: PLoS One. 2012 Jan 20;7(1):e30421. doi: 10.1371/journal.pone.0030421 (PMC3262810; doi:10.1371/journal.pone.0030421)
Supplement: Table S1 — Strains and plasmids used in this study. (PDF) [file pone.0030421.s001.pdf]

Table S1. Strains and plasmids used in this study.

Strains / Plasmids	Description	Reference or source
**Strains:**		
Azoarcus sp.		
BH72	wild type	[1]
BHΔ*pilS*	BH72 carrying in frame deletion of *pilS*	This study
BH*pilS*M	point mutation of *pilS*, His314Arg	This study
BH72::pJBLP14	Ap^r, *pilAB*::*uidA*	This study
BHΔ*pilS*::pJBLP14	Ap^r, pJBLP14 chromosomally integrated in BHΔ*pilS*	This study
BHΔ*pilS*::pJBLP1*gfp*	Ap^r, *pilAB::gfp*	This study
BH*pilS*M::pJBLP14	Ap^r, pJBLP14 chromosomally integrated in BH*pilS*M	This study
BHΔ0390	Km^r, insertion *azo0390*::pK0390	This study
BHΔ1746	Km^r, insertion *azo1746*::pK1746	This study
BHΔ3178	Km^r, insertion *azo3178*::pK3178	This study
BHΔ3379	Km^r, insertion *azo3379*::pK3379	This study
BH*azo1544*	Km^r, plasmid pK18GGST*azo1544* chromosomally integrated into the genome of *Azoarcus* sp. BH72, *azo1544*::*gfp*::*uidA*-fusion	Teja Shidore, this study
BH*azo1684*	Km^r, plasmid pK18GGST-*1684*pro chromosomally integrated into the genome of *Azoarcus* sp. BH72, *azo1684*::*gfp*::*uidA*-fusion	This study
BH*azo2876*	Km^r, plasmid pK18GGST-*2876*pro chromosomally integrated into the genome of *Azoarcus* sp. BH72, *azo2876*::*gfp*::*uidA*-fusion	This study
BH*azo3874*	Km^r, plasmid pK18GGST*azo3874* chromosomally integrated at position 4246383 of *Azoarcus* sp. BH72 genome, *azo3874*::*gfp*::*uidA*-fusion	This study
A. communis SWub3	wild type	[2]
A. evansii KB740	wild type	[3]
Chromobacterium violaceum		
ATCC31532	wild type	
CV026	Km^r, mini-Tn5 mutant of ATCC31532, AHL^-	[4]
CV017	mini-Tn5 double mutant of ATCC31532, overproduction of violacein	Williams, unpublished
Pseudomonas sp.		
P. putida IsoF (pKR-C12)	wild type, plasmid Gm_r, pBBR1MCS-5 carrying P_{lasB}*gfp*(ASV) P_{lac}-*lasR*	[5]
P. syringae pv. tomato DC3000	wild type	M. Ullrich, Jacobs Univ.
P. stutzeri DSM4166	wild type	DSMZ
Rhizobium sp. NGR234	wild type, New Guinea isolate	[6]
Azospirillum lipoferum Sp59b	wild type	[7]
Azospirillum brasilense Sp7	wild type	[7]
Azonexus fungiphilus Bs5-8	wild type	[8]
Azovibrio restrictus S5b2	wild type	[2]
Azotobacter vinelandii MV521	wild type	[9]
Xanthomonas oryzae PXO99	wild type	C. VeraCruz, IRRI
**Plasmids:**		
pJBLP14	Ap^r, derivative of pUC19 plasmid, with 1.84 kb *uidA*-fragment and *pilAB* on a 5.4 kb chromosomal	[10]

	BamHI-fragment of strain BH72, contains *pilAB*::*uidA*-fusion	
pJBLP1*gfp*	Ap^{r}, 0,7 kb *Eco*RI fragment of pSKGFP in *Mfe*I site of pJBLP1, *pilAB::gfp* fusion	This study
pJBLP1	Ap^{r}, insert of pJBL1 cloned in pUC19	[11]
pUC19	Ap^{r}, ColE1 origin	[12]
pSKGFP	Ap^{r}, 0.7 kb *gfp* fragment in pBluescript SK(+/-)	[13]
pK390	Km^{r}, 0.55 kb *Hind*III-*Xba*I fragment of pPCR390 in *Hind*III-*Xba*I site of pK18*mob*2	This study
pK1746	Km^{r}, 0.56 kb *Hind*III-*Xba*I fragment of pPCR1746 in *Hind*III-*Xba*I site of pK18*mob*2	This study
pK3178	Km^{r}, 0.58 kb *Hind*III-*Xba*I fragment of pPCR3178 in *Hind*III-*Xba*I site of pK18*mob*2	This study
pK3379	Km^{r}, 0.57 kb *Hind*III-*Xba*I fragment of pPCR3379 in *Hind*III-*Xba*I site of pK18*mob*2	This study
pK18*mob*2	Km^{r}, pK18*mob* derivative with unique *Kpn*I and *Sac*I sites in polylinker	[14]
pK18*mobsacB*	Km^{r}, RP4 mob region, containing *sacB* gene	[15]
pK18GGST	Km^{r}, derivative of the mobilizable cloning vector pK18mob2, promoterless *gfp* and *uidA*, T4 transcription terminator	A. Krause, unpublished
pK18GGST*azo1544*	Km^{r}, fragment of *azo1544* at *Xba*I-*Hind*III site in pK18GGST (88-694 bp of gene *azo1544*)	Teja Shidore, this study
pK18GGST-*1684*pro	Km^{r}, fragment upstream of *azo1684* (bp 399/ *azo1685* to bp 133/ *azo1684*) at *Hin*dII-*Xba*I site in pK18GGST	This study
pK18GGST-*2876*pro	Km^{r}, fragment upstream of *azo2876* (bp 172/ *azo2875* to bp 368/ *azo2876* at *Sph*I-*Hin*dIII site in pK18GGST	This study
pK18GGST*azo3874*	Km^{r}, fragment of *azo3874* at HindIII/ XbaI site in pK18GGST (14-470 bp of gene *azo3874*)	This study
pJBLP2	Ap^{r}, *Eco*RI-*Sst*I fragment of pJBL2 in pUC19, containing *pilSR*	[10]
pJBLP23	Ap^{r}, 4.3 kb *Xho*I-*Asp*718 fragment of pJBLP2 in pUC19	This study
pJBLP231	Ap^{r}, 1.8 kb *Sma*I fragment of pJBLP23 in pUC19	This study
pJBLP2311	Ap^{r}, deletion of 0.9 kb *Nru*I-*Bsa*BI fragment from pJBLP231	This study
pJBLP232	Ap^{r}, exchange of 1.8 kb *Sma*I fragment of pJBLP23 against 0.9 kb *Sma*I fragment of pJBLP2311	This study
pJBLP234	Km^{r} , 3.2 kb *Eco*RI-*Hind*III fragment of pJBLP232 in pK18*mobsacB*	This study

1. Reinhold B, Hurek T, Niemann E-G, Fendrik I (1986) Close association of *Azospirillum* and diazotrophic rods with different root zones of Kallar grass. Appl. Environ Microbiol 52:520-526.
2. Reinhold-Hurek B, Hurek T, Gillis M, Hoste B, Vancanneyt M, Kersters K, De Ley J (1993) *Azoarcus* gen. nov., nitrogen-fixing proteobacteria associated with roots of Kallar grass

(*Leptochloa fusca* (L.) Kunth) and description of two species *Azoarcus indigens* sp. nov. and *Azoarcus communis* sp. nov. Int J Syst Bacteriol 43:574-584.

3. Anders HJ, Kaetzke A, Kämpfer P, Ludwig W, Fuchs G (1995) Taxonomic position of aromatic-degrading denitrifying pseudomonad strains K 172 and KB 740 and their description as new members of the genera *Thauera*, as *Thauera aromatica* sp. nov., and *Azoarcus*, as *Azoarcus evansii* sp. nov., respectively, members of the beta subclass of the *Proteobacteria*. Int J Syst Bacteriol 45:327-333.
4. McClean KH, Winson MK, Fish L, Taylor A, Chhabra SR, Camara M, Daykin M, Lamb JH, Swift S, Bycroft BW, Stewart GS, Williams P (1997) Quorum sensing and *Chromobacterium violaceum*: exploitation of violacein production and inhibition for the detection of *N*-acylhomoserine lactones. Microbiol 143:3703-3711.
5. Riedel K, Hentzer M, Geisenberger O, Huber B, Steidle A, Wu H, Hoiby N, Givskov M, Molin S, Eberl L (2001) N-acylhomoserine-lactone-mediated communication between *Pseudomonas aeruginosa* and *Burkholderia cepacia* in mixed biofilms. Microbiol 147:3249-3262.
6. Trinick MJ (1980) Relationships amongst the fast-growing rhizobia of *Lablab purpureus*, *Leucaena leucocephela, Mimosa* spp., *Acacia farnesiana* and *Sesbania grandiflora* and their affinities with other rhizobial groups. J Appl Bacteriol 49:39-53.
7. Tarrand JJ, Krieg NR, Döbereiner J (1978) A taxonomic study of the *Spirillum lipoferum* group, with description of a new genus, *Azospirillum* gen. nov. and two species, *Azospirillum lipoferum* (Beijerinck) comb. nov. and *Azospirillum brasilense* sp. nov. Can J Microbiol 24:967-980.
8. Hurek T, Wagner B, Reinhold-Hurek B (1997) Identification of N_2-fixing plant- and fungus-associated *Azoarcus* species by PCR-based genomic fingerprints. Appl Environ Microbiol 63:4331-4339.
9. Walmsley J, Toukdarian A, Kennedy C (1994) The role of regulatory genes *nifA, vnfA, anfA, nfrX, ntrC*, and *rpoN* in expression of genes encoding the three nitrogases of *Azotobacter vinelandii*. Arch Microbiol 162:422-429.
10. Hauberg L, Schmidt F, Scharf C, Dörr J, Völker U, Reinhold-Hurek B (2010) Proteomic characterization of a *pilR* regulatory mutant of *Azoarcus* sp. strain BH72 with the aid of gel-based and gel-free approaches. Proteomics 10:458-469.
11. Dörr J, Hurek T, Reinhold-Hurek B (1998) Type IV pili are involved in plant-microbe and fungus-microbe interactions. *Molecular Microbiology* 30:7-17.
12. Yanisch-Perron C, Vieira J, Messing J (1985) Improved M13 phage cloning vectors and host strains: nucleotide sequences of the M13mp18 and pUC19 vectors. Gene 33:103-119.
13. Egener T, Hurek T, Reinhold-Hurek B (1998) Use of green fluorescent protein to detect expression of *nif* genes of *Azoarcus* sp. BH72, a grass-associated diazotroph, on rice roots. MPMI 11:71-75.
14. Tauch A, Zheng Z, Pühler A, Kalinowski J (1998) *Corynebacterium striatum* chloramphenicol resistance transposon Tn5564: genetic organization and transposition in *Corynebacterium glutamicum*. Plasmid 40:126-139.
15. Schäfer A, Tauch A, Jäger W, Kalinowski J, Thierbach G, Pühler A (1994) Small mobilizable multi-purpose cloning vectors derived from the *Escherichia coli* plasmids pK18 and pK19: selection of defined deletions in the chromosome of *Corynebacterium glutamicum*. Gene 145:69-73.
