# Supplementary material for: Environmental Factors Affecting the Expression of pilAB as Well as the Proteome and Transcriptome of the Grass Endophyte Azoarcus sp. Strain BH72
Source: PLoS One. 2012 Jan 20;7(1):e30421. doi: 10.1371/journal.pone.0030421 (PMC3262810; doi:10.1371/journal.pone.0030421)
Supplement: Table S2 — Characterization of the autoinducer molecule in conditioned supernatant, determined as pilAB :: uidA -inducing activity in supernatant bioassays with strain Azoarcus sp. BHΔ pilS ::pJBLP14a. (PDF) [file pone.0030421.s002.pdf]

Table S2. Characterization of the autoinducer molecule in conditioned supernatant, determined as *pilAB*::*uidA*-inducing activity in supernatant bioassays with strain *Azoarcus* sp. BHΔ*pilS*::pJBLP14 [a]

Sample treatment	*pilAB* - inducing activity: Fold induction (± standard deviation)	Activity retained after treatment (%)
Dichloromethane extraction		
conditioned supernatant (untreated)	1.75 ± 0.09 [b]	100
aqueous phase of the dichloromethane extraction	1.50 ± 0.07 [b]	86
dichloromethane extract	0.90 ± 0.05	51
Mutant analysis		
Azoarcus BH72	2.01 ± 0.23 [c]	
Azoarcus BHΔ0390	2.39 ± 0.10 [c]	
Azoarcus BHΔ1746	2.17 ± 0.31 [c]	
Azoarcus BHΔ3178	2.28 ± 0.17 [c]	
Azoarcus BHΔ3379	2.23 ± 0.53 [c]	

[a] Same results were obtained from two to three independent experiments with two to four repetitions.

[b] Induction factors significantly different (P< 0.05) to the uninduced control (one sample t-test).

[c] Induction factors significantly different (P< 0.01) to the uninduced control (one sample t-test), from two independent experiments.
