# Supplementary material for: Environmental Factors Affecting the Expression of pilAB as Well as the Proteome and Transcriptome of the Grass Endophyte Azoarcus sp. Strain BH72
Source: PLoS One. 2012 Jan 20;7(1):e30421. doi: 10.1371/journal.pone.0030421 (PMC3262810; doi:10.1371/journal.pone.0030421)
Supplement: Table S3 — Differentially expressed genes detected by microarray analyses in Azoarcus sp. BH72 upon incubation in conditioned supernatant. (PDF) [file pone.0030421.s003.pdf]

Table S3. Differentially expressed genes detected by microarray analyses in *Azoarcus* sp. BH72 upon incubation in conditioned supernatant

Acc. No.[a)]	fold[b)] 1h	fold[b)] 4h	Gene	Product Name	P-value[b)] 1h	P-value[b)] 4h	*pilR*-mutant[c)] (BH*pilR*K)
azo0018	-1.02	2.46		ThiJ/PfpI family protein	0.265	0.011	
azo0086*	-1.90	-1.95	*efp*	translation elongation factor P (EF-P)	0.006	0.007	
azo0092	1.54	2.13	*mucD1*	probable serine protease	0.169	0.034	
azo0099	1.95	1.15	*def1*	peptide deformylase	0.000	0.034	
azo0119	1.56	3.23		amino acid/amide ABC transporter substrate-binding protein, HAAT family	0.030	0.043	
azo0152	1.11	-2.11		conserved hypothetical membrane protein	0.127	0.002	
azo0153	1.11	-1.92	*atpB*	probable ATP synthase A chain	0.004	0.010	
azo0154	-1.39	-2.45	*atpE*	ATP synthase F0 subcomplex C subunit	0.116	0.001	
azo0155	-2.21	-2.45	*atpF*	ATP synthase F0 subcomplex B subunit	0.155	0.006	
azo0156*	-2.37	-2.53	*atpH*	ATP synthase F1 subcomplex delta subunit	0.017	0.022	down
azo0157	-2.19	-1.84	*atpA*	ATP synthase F1 subcomplex alpha subunit	0.05	0	
azo0158	-2.55	-2.08	*atpG*	ATP synthase F1 subcomplex gamma subunit	0.021	0.011	
azo0159	-1.48	-2.77	*atpD*	ATP synthase beta chain	0.038	0.004	
azo0160	-1.75	-2.07	*atpC*	ATP synthase F1 subcomplex epsilon subunit	0.024	0.047	down
azo0163	1.67	2.00		conserved hypothetical protein	0.013	0.003	
azo0165	1.58	3.13	*phoR*	PAS/PAC sensor signal transduction histidine kinase	0.220	0.017	
azo0247	1.89	1.15	*bfr1*	putative bacterioferritin	0.006	0.044	
azo0275	3.75	1.61		conserved hypothetical secreted protein	0.045	0.064	
azo0294	-1.93	-2.07		putative penicillin-binding protein	0.030	0.015	
azo0300	-2.44	1.92	*paaG1*	probable enoyl-CoA hydratase	0.016	0.049	
azo0303	-2.18	1.86	*paaK*	phenylacetate-CoA ligase	0.002	0.024	
azo0304	-1.57	1.89	*paaA*	phenylacetic acid degradation protein	0.009	0.050	
azo0305	-1.98	1.65	*paaB*	phenylacetic acid degradation protein	0.010	0.102	down
azo0307	-1.83	1.84	*paaD*	probable phenylacetic acid degradation protein	0.005	0.001	
azo0308	-1.84	1.43	*paaE*	probable phenylacetic acid degradation NADH oxidoreductase	0.002	0.109	
azo0321	1.90	1.85	*cutA2*	putative protein disulfide-isomerase	0.096	0.003	
azo0347	1.96	2.86		hypothetical secreted protein	0.029	0.037	
azo0352	2.12	2.09		conserved hypothetical protein	0.001	0.009	
azo0368	-1.20	-1.99	*mmpI*	probable immunodominant 35kDa protein	0.232	0.013	
azo0386	4.01	1.93		hypothetical membrane protein	0.038	0.293	
azo0391	1.22	2.01		putative MerR-family transcriptional regulator	0.222	0.037	
azo0430	1.11	-3.53	*cysH*	phosphoadenylylsulfate reductase (thioredoxin)	0.132	0.003	
azo0432	-1.17	-2.99	*cysI*	putative sulfite reductase	0.180	0.004	
azo0438	2.15	-1.09	*ansB2*	periplasmic L-asparaginase II	0.004	0.049	up
azo0440	1.86	-1.09	*glnM*	amino acid ABC transporter membrane protein 2, PAAT family	0.002	0.160	
azo0456	13.30	13.06		hypothetical secreted protein	0.021	0.002	
azo0457	-1.82	-1.89	*ragA*	two component transcriptional regulator, winged helix family	0.023	0.007	
azo0488	2.05	1.84		conserved hypothetical protein	0.012	0.004	
azo0499	1.97	1.98	*pheC*	amino acid ABC transporter substrate-binding protein, PAAT family	0.023	0.002	
azo0536	-1.44	-3.15		conserved hypothetical protein	0.017	0.006	
azo0544	-1.00	-2.13		hypothetical protein	0.199	0.015	
azo0586	-2.05	-2.07		Hypothetical protein	0.004	0.089	
azo0587	-3.08	-1.81	*acyH*	adenosylhomocysteinase	0.004	0.055	down
azo0590	-2.31	-2.16	*htrB*	putative lipid A biosynthesis lauroyl acyltransferase	0.232	0.033	

azo0616	1.88	2.00	*bfr2*	putative bacterioferritin	0.006	0.032	down
azo0617	1.86	1.84	*bfd*	conserved hypothetical bacterioferritin-associated ferredoxin	0.005	0.015	
azo0622	1.87	2.18		diguanylate cyclase/phosphodiesterase	0.063	0.009	
azo0644	2.08	1.39		putative regulatory protein	0.012	0.007	
azo0649	2.33	2.12		conserved hypothetical protein	0.018	0.000	
azo0650	1.66	2.01		conserved hypothetical glycosyltransferase	0.047	0.019	
azo0659	1.00	2.14	*gor*	NADPH-glutathione reductase	0.127	0.018	
azo0669	2.18	2.66	*napC1*	periplasmic nitrate reductase subunit	0.033	0.005	
azo0670	1.07	1.83	*napB1*	periplasmic nitrate reductase subunit	0.103	0.003	
azo0671	1.62	2.81	*napA1*	periplasmic nitrate reductase subunit apoprotein	0.034	0.035	
azo0672	1.07	3.51	*napD1*	putative NapD protein	0.070	0.026	
azo0673	2.72	4.60	*napE*	putative periplasmic nitrate reductase accessory protein	0.030	0.005	
azo0682	-2.05	-2.74		conserved hypothetical sodium:solute symporter	0.005	0.017	
azo0717	1.27	3.28		conserved hypothetical secreted protein	0.147	0.015	
azo0718*	-1.52	-2.36	*rpsF*	SSU ribosomal protein S6P	0.074	0.015	
azo0719	-3.03	-3.23	*priB*	putative primosomal replication protein	0.020	0.007	
azo0720	-1.17	-1.91	*rpsR*	SSU ribosomal protein S18P	0.052	0.002	
azo0746	-3.16	-2.25		hypothetical membrane protein	0.072	0.007	
azo0753	-1.43	-2.54	*pth*	peptidyl-tRNA hydrolase	0.135	0.019	
azo0754*	1.02	-1.97	*rplY*	LSU ribosomal protein L25P	0.061	0.013	
azo0755	-1.81	-2.65	*prsA*	Ribose-phosphate diphosphokinase	0.118	0.010	
azo0864	-1.10	-2.27	*accB*	biotin carboxyl carrier protein	0.068	0.019	
azo0865	-1.17	-1.88	*aroQ*	3-dehydroquinate dehydratase	0.221	0.012	
azo0867	1.95	1.75		conserved hypothetical protein	0.008	0.068	
azo0875	-1.70	-2.96	*mraZ*	protein mraZ	0.067	0.026	
azo0898	1.42	2.35		putative serine/threonine protein kinase	0.236	0.022	
azo0899	1.81	2.28		hypothetical membrane protein	0.020	0.012	
azo0923	-1.15	-2.51	*ndk*	nucleoside diphosphate kinase	0.056	0.003	up
azo0974	-2.10	-1.82	*groEL1*	chaperonin	0.002	0.084	down
azo1062*	-1.20	-2.65	*dnaJ1*	chaperone protein	0.173	0.001	down
azo1064	-1.24	-2.84	*grpE*	probable heat shock protein	0.062	0.017	down
azo1072	-2.11	-2.32	*rpsA*	SSU ribosomal protein S1P	0.041	0.013	
azo1073	-1.51	-2.52	*ihfB*	probable integration host factor, beta-subunit	0.006	0.003	
azo1080	-2.05	-2.55	*infC*	bacterial translation initiation factor 3 (bIF-3)	0.047	0.005	
azo1081	-1.30	-2.04	*rpmI*	LSU ribosomal protein L35P	0.183	0.014	
azo1082	-2.64	-3.37	*rplT*	LSU ribosomal protein L20P	0.004	0.001	
azo1090	1.83	2.14	*rpoS*	RNA polymerase, sigma 38 subunit	0.080	0.009	
azo1117	-1.82	-2.20	*aceA*	isocitrate lyase	0.005	0.005	
azo1135	-1.87	-2.99	*rpmG*	LSU ribosomal protein L33P	0.021	0.004	
azo1159	-2.36	1.23	*aceB*	AceB protein	0.014	0.184	
azo1193	1.57	2.07	*hppD*	probable 4-hydroxyphenylpyruvate dioxygenase	0.075	0.002	
azo1212	-1.86	-2.59		conserved hypothetical protein	0.026	0.092	
azo1222	1.92	1.16	*dmpM*	phenol 2-monooxygenase	0.002	0.001	
azo1226	-2.09	-2.47	*lguL*	lactoylglutathione lyase	0.043	0.018	
azo1272	2.77	2.12		conserved hypothetical secreted protein	0.254	0.006	
azo1277	1.87	2.30		conserved hypothetical glutathione peroxidase	0.059	0.006	down
azo1280*	-1.47	-5.39	*fpr1*	ferredoxin-NADP+ reductase	0.061	0.018	
azo1285	-2.03	1.13	*gcvP*	glycine dehydrogenase (decarboxylating) alpha subunit / beta subunit	0.018	0.105	
azo1286	-2.39	1.18	*gcvH*	glycine cleavage system H protein	0.010	0.180	
azo1345	1.29	1.83	*ccoG*	putative iron-sulfur 4Fe-4S ferredoxin transmembrane protein	0.221	0.001	
azo1349	2.29	2.10		putative universal stress protein f	0.004	0.020	
azo1350	2.04	3.13	*phbC2*	probable poly-beta-hydroxybutyrate synthase	0.009	0.013	

azo1368	2.57	7.54		conserved hypothetical protein	0.254	0.004	
azo1377	-1.47	-2.04	*folD*	5,10-methylenetetrahydrofolate dehydrogenase (NADP+) / methenyltetrahydrofolate cyclohydrolase	0.145	0.013	
azo1396	-1.23	-3.33	*nuoA*	NADH dehydrogenase subunit A	0.008	0.006	
azo1397	-1.82	-2.49	*nuoB2*	NADH dehydrogenase subunit B	0.123	0.006	
azo1399	-1.38	-2.21	*nuoD*	NADH dehydrogenase subunit D	0.169	0.011	
azo1400	-1.59	-2.26	*nuoE*	NADH dehydrogenase subunit E	0.004	0.013	
azo1401	-2.12	-2.21	*nuoF*	NADH dehydrogenase subunit F	0.027	0.008	
azo1403	-2.28	-2.11	*nuoH*	NADH dehydrogenase subunit H	0.012	0.128	
azo1404	-2.19	-2.45	*nuoI*	NADH dehydrogenase subunit I	0.014	0.001	
azo1406	-2.72	-2.80	*nuoK*	NADH dehydrogenase subunit K	0.020	0.004	
azo1407	-1.53	-2.22	*nuoL*	NADH dehydrogenase subunit L	0.017	0.001	
azo1408	-1.10	-2.26	*nuoM*	NADH dehydrogenase subunit M	0.067	0.002	
azo1442	1.51	2.04		putative nuclease	0.097	0.014	
azo1468	-1.12	-2.17	*exbB3*	conserved hypothetical biopolymer transport protein	0.156	0.023	
azo1497	-1.16	-2.14	*prfB*	bacterial peptide chain release factor 2 (bRF-2)	0.149	0.004	
azo1521	-1.13	-2.43	*rpmE*	LSU ribosomal protein L31P	0.235	0.003	
azo1544	1.83	1.98		diguanylate cyclase/phosphodiesterase with PAS/PAC sensor(s)	0.001	0.011	
azo1586	2.14	1.29		conserved hypothetical secreted protein	0.017	0.156	
azo1608	3.37	3.08		hypothetical protein	0.164	0.002	
azo1620	-2.22	-2.30		conserved hypothetical protein	0.008	0.028	
azo1621	1.16	-2.57	*rpmF*	LSU ribosomal protein L32P	0.181	0.099	
azo1623	-1.33	-2.87	*fabH*	3-oxoacyl-[acyl-carrier-protein] synthase III	0.064	0.008	
azo1630	1.15	1.96	*algU*	RNA polymerase, sigma-24 subunit	0.173	0.023	
azo1652	1.29	1.88		conserved hypothetical protein	0.187	0.018	
azo1654	-1.19	-1.95		cell division topological specificity factor MinE	0.242	0.002	
azo1659	1.07	-2.05	*tex*	transcription accessory protein	0.225	0.022	
azo1675	2.22	2.10		putative phosphoribosyltransferase	0.013	0.001	
azo1684	1.86	10.85		conserved hypothetical protein	0.001	0.001	
azo1694	1.81	1.83		conserved hypothetical protein	0.024	0.009	
azo1699	-2.48	-1.39	*etfB1*	electron transfer flavoprotein, beta subunit	0.008	0.005	up
azo1700	-2.58	-1.29	*etfA1*	probable electron transfer flavoprotein, alpha subunit	0.001	0.007	
azo1701	-2.14	-1.86		conserved hypothetical membrane protein	0.149	0.011	
azo1715	2.22	1.10	*rbcR*	transcriptional regulator	0.015	0.150	
azo1727	1.97	1.82	*treS*	trehalose synthase	0.035	0.011	
azo1743	1.00	2.59		conserved hypothetical protein	0.300	0.008	
azo1851	2.17	1.82		conserved hypothetical protein	0.004	0.019	
azo1864	1.88	2.14		Hypothetical protein	0.020	0.093	
azo1874	1.86	1.28	*rmlC*	dTDP-4-dehydrorhamnose 3,5-epimerase	0.032	0.064	
azo1875	1.88	1.17	*rmlA*	Glucose-1-phosphate thymidylyltransferase	0.009	0.275	
azo1877	1.86	1.33	*rmlB*	dTDP-glucose 4,6-dehydratase	0.012	0.089	
azo1887	1.94	1.28		conserved hypothetical protein	0.008	0.048	
azo1896	1.33	-1.90	*lpxB*	lipid-A-disaccharide synthase	0.102	0.016	
azo1908	-2.19	-2.47	*tsf*	translation elongation factor Ts (EF-Ts)	0.070	0.007	up
azo1909	-1.26	-3.47		SSU ribosomal protein S2P	0.040	0.024	
azo1918	2.23	2.48		conserved hypothetical secreted protein	0.115	0.020	
azo1922	-2.15	-1.29	*etfB2*	electron transfer flavoprotein, beta-subunit	0.014	0.004	down
azo1948	-1.40	-1.89	*padD*	phenylacetyl-CoA:acceptor oxidoreductase PadD subunit	0.172	0.015	
azo1965	1.19	1.89		transcriptional regulator, AraC family	0.270	0.004	
azo1978	1.98	1.93		conserved hypothetical protein	0.028	0.025	
azo2008	1.89	1.44		[LSU ribosomal protein L3P]-glutamine N5-methyltransferase	0.005	0.034	
azo2062*	1.24	2.32		conserved hypothetical peptidyl-prolyl cis-trans isomerase	0.239	0.026	down

azo2063	1.20	2.06	*bcp1*	putative bacterioferritin comigratory protein	0.110	0.014	
azo2070	1.82	-1.12	*clpX*	ATP-dependent Clp protease ATP-binding subunit	0.011	0.285	
azo2072	-1.03	-2.93	*tig*	trigger factor	0.056	0.006	
azo2073	1.99	4.33	*prkA*	putative serine protein kinase	0.021	0.007	
azo2074	2.64	4.49	*yeaH*	conserved hypothetical protein	0.039	0.004	
azo2075	2.05	2.42	*ycgB*	putative cytoplasmic protein	0.013	0.014	
azo2103	-1.47	-2.31	*pnp*	polyribonucleotide nucleotidyltransferase	0.055	0.025	
azo2104	-1.42	-2.38	*rpsO*	SSU ribosomal protein S15P	0.041	0.009	
azo2109	-1.24	-2.31		conserved hypothetical protein	0.039	0.021	
azo2120	1.00	-2.12	*ylqF*	probable GTPase	0.275	0.012	
azo2121	-2.03	-2.85	*cspA*	cold-shock DNA-binding protein family	0.007	0.001	
azo2142	1.58	4.14		putative inosine-5'-monophosphate dehydrogenase related protein	0.027	0.012	
azo2148	1.25	1.95	*sndH*	putative L-sorbosone dehydrogenase	0.119	0.014	
azo2151	1.09	-1.97	*etf1*	probable electron transfer flavoprotein-ubiquinone oxidoreductase	0.261	0.022	up
azo2156	2.42	-1.32		probable TonB-dependent receptor	0.017	0.037	up
azo2175	1.79	2.43	*pilY1A*	putative type 4 pilus biogenesis protein	0.111	0.001	
azo2177	1.28	2.06	*pilW*	putative type 4 pilus biogenesis protein	0.047	0.012	
azo2180	1.10	3.00		conserved hypothetical prepilin like protein	0.171	0.003	
azo2186	-1.93	-1.94	*argG*	argininosuccinate synthase	0.008	0.003	
azo2190	-1.28	-2.27	*rpsT*	SSU ribosomal protein S20P	0.255	0.010	
azo2192	-1.81	-2.22		Hypothetical protein	0.022	0.151	
azo2197	2.24	1.84	*bcp2*	putative bacterioferritin comigratory protein	0.042	0.025	
azo2220	2.97	1.95	*cbiM*	putative cobalt transport system, permease protein	0.012	0.030	
azo2224	-1.11	-2.38	*yaiI*	YaiI/YqxD family protein	0.110	0.003	
azo2257	-1.11	-2.32	*mauA*	probable methylamine dehydrogenase, L chain	0.344	0.018	
azo2290	2.06	1.51		conserved hypothetical protein	0.026	0.043	
azo2314	1.35	2.67		glycosyltransferase	0.239	0.007	
azo2324	2.43	2.47		putative polysaccharide deacetylase	0.052	0.004	
azo2396*	-1.73	-2.00		putative TonB-dependent receptor	0.008	0.025	down
azo2405	-1.75	-2.18	*ohr*	probable organic hydroperoxide resistance protein	0.040	0.006	
azo2408	9.75	2.24		hypothetical sensor protein	0.008	0.033	
azo2442	2.02	2.00	*poxC*	phenol 2-monooxygenase P2 subunit	0.152	0.007	
azo2469	1.00	2.63		conserved hypothetical protein	0.202	0.009	
azo2492	-1.81	-1.87	*etfB3*	electron transfer flavoprotein, beta subunit	0.019	0.012	
azo2552	1.10	2.41	*pilU1*	twitching motility protein	0.285	0.000	
azo2561	1.96	3.91		conserved hypothetical protein	0.086	0.018	
azo2563	1.90	2.27		conserved hypothetical secreted protein	0.006	0.002	
azo2588	-2.38	1.15	*ompA1*	outer membrane protein A precursor	0.006	0.037	
azo2640	-1.37	2.71	*mdcH*	putative transcriptional factor	0.134	0.001	
azo2646	1.71	2.48		conserved hypothetical protein	0.076	0.019	
azo2651	2.66	2.53		conserved hypothetical protein	0.002	0.017	
azo2656	1.53	2.50		conserved hypothetical membrane protein	0.086	0.006	
azo2664	-1.28	2.41		conserved hypothetical protein	0.280	0.019	
azo2672	1.50	1.84		conserved hypothetical sensor histidine kinase	0.128	0.019	
azo2690	1.08	2.35		conserved hypothetical protein	0.162	0.008	
azo2694	1.89	2.00		nucleotide sugar aminotransferase	0.146	0.036	
azo2698	2.71	1.93	*pepM*	putative phosphoenolpyruvate phosphomutase	0.126	0.004	
azo2758	2.16	2.72		conserved hypothetical protein	0.001	0.001	
azo2759	-2.03	-3.16	*rpsI*	SSU ribosomal protein S9P	0.014	0.011	
azo2760	-1.35	-2.99	*rplM*	LSU ribosomal protein L13P	0.016	0.004	
azo2763	-1.09	-2.08		conserved hypothetical secreted protein	0.137	0.007	
azo2790	2.24	2.82		conserved hypothetical protein	0.017	0.017	
azo2813	-1.95	-1.65		conserved hypothetical secreted protein	0.008	0.006	
azo2827	2.03	1.76	*phoU*	phosphate uptake regulator	0.018	0.030	

azo2835	1.91	1.39	*prkB*	probable phosphoribulokinase	0.009	0.143	
azo2844	1.00	2.32	*exaA1*	putative quinoprotein ethanol dehydrogenase	0.066	0.018	
azo2845	1.00	2.03	*qbdB2*	conserved hypothetical secreted protein	0.125	0.001	
azo2871	2.14	5.46		conserved hypothetical YceI like protein	0.001	0.017	
azo2876	3.33	6.82		conserved hypothetical membrane protein	0.013	0.014	
azo2883	1.93	2.02		conserved hypothetical secreted protein	0.149	0.007	
azo2898	-1.09	-3.06	*rpsP*	SSU ribosomal protein S16P	0.033	0.002	
azo2901	-2.04	-2.07	*rplS*	LSU ribosomal protein L19P	0.021	0.001	
azo2914	1.88	1.41	*pilV*	putative prepilin-like protein	0.033	0.022	
azo2916	3.41	2.16	*pilX*	putative Tfp pilus assembly protein	0.017	0.016	
azo2956	-1.16	2.55		putative cooper-transporting ATPase protein	0.254	0.009	
azo2969	1.95	2.21		conserved hypothetical secreted protein	0.091	0.024	
azo2977	1.10	1.92	*cphA*	putative beta lactamase precursor	0.126	0.011	
azo2987	1.57	2.32		dihydrofolate reductase, putative	0.131	0.016	
azo3023	-5.24	-1.15		probable TonB-dependent receptor	0.001	0.010	
azo3041	2.21	2.41	*rubA*	probable rubredoxin	0.017	0.021	
azo3047	1.98	1.12	*livG1*	amino acid/amide ABC transporter ATP-binding protein 1, HAAT family	0.025	0.152	
azo3050	1.00	4.26	*livJ*	amino acid/amide ABC transporter substrate-binding protein, HAAT family	0.322	0.007	up
azo3060	3.79	2.42		conserved hypothetical protein	0.022	0.023	
azo3073	2.47	1.49		hypothetical protein	0.027	0.176	
azo3128	2.15	2.13	*nikR*	transcriptional regulator, CopG family	0.019	0.012	
azo3146	1.00	3.08		conserved hypothetical protein	0.021	0.001	
azo3160	3.09	3.62		conserved hypothetical protein	0.087	0.020	
azo3167	-1.08	-2.30	*rplU*	LSU ribosomal protein L21P	0.203	0.001	
azo3168	-1.23	-3.25	*rpmA*	LSU ribosomal protein L27P	0.097	0.016	
azo3194	1.41	2.54		conserved hypothetical protein	0.172	0.035	
azo3212	1.60	1.93	*parA3*	ParA family protein	0.007	0.002	
azo3225	-1.11	-1.82	*rpsU*	SSU ribosomal protein S21P	0.014	0.018	
azo3287	1.14	2.01		conserved hypothetical secreted protein	0.319	0.015	
azo3293	2.42	3.42	*senC*	SCO1/SenC family protein	0.004	0.001	
azo3294	5.07	11.34	*coxD*	4-hydroxybenzoate octaprenyltransferase	0.022	0.001	
azo3300	-1.87	-2.61		conserved hypothetical membrane protein	0.228	0.022	
azo3303	1.00	1.82	*coxA*	probable cytochrome c oxidase, subunit I	0.099	0.019	
azo3304	1.50	2.10	*coxB*	conserved hypothetical cytochrome c oxidase, subunit II	0.194	0.008	
azo3319	1.93	1.25	*fkbP*	peptidyl-prolyl cis-trans isomerase	0.009	0.018	
azo3320	1.88	2.14	*msrA*	putative peptide methionine sulfoxide reductase	0.026	0.026	
azo3325	-1.98	-2.17	*trpE*	anthranilate synthase, component I	0.089	0.009	
azo3327	1.28	-1.81	*rpe*	ribulose-5-phosphate 3-epimerase	0.203	0.001	
azo3330	2.10	1.83		FHA-domain containing protein	0.005	0.078	
azo3354**	1.58	1.74	*pilB*	Type IV pilus assembly protein	0.157	0.048	down
azo3355	1.38	2.98	*pilA*	tfp structural protein	0.133	0.025	down
azo3356	1.97	1.38	*pilR*	Two-component response regulator	0.009	0.239	
azo3364	-1.57	-1.92		glycosyl transferase	0.081	0.009	
azo3388	2.03	3.16		hypothetical protein	0.006	0.002	
azo3390	-2.12	-2.27	*rplQ*	LSU ribosomal protein L17P	0.013	0.013	
azo3391	-3.38	-2.22	*rpoA*	DNA-directed RNA polymerase subunit alpha	0.010	0.041	
azo3393	-2.75	-2.02	*rpsK*	SSU ribosomal protein S11P	0.002	0.004	
azo3394	-1.55	-2.20	*rpsM*	SSU ribosomal protein S13P	0.098	0.016	
azo3395	-1.24	-2.05	*rpmJ*	LSU ribosomal protein L36P	0.158	0.010	
azo3398	-1.73	-2.16	*rplO*	LSU ribosomal protein L15P	0.110	0.016	
azo3399	-2.10	-2.07	*rpmD*	LSU ribosomal protein L30P	0.193	0.000	
azo3400	-2.34	-2.03	*rpsE*	SSU ribosomal protein S5P	0.007	0.003	
azo3401	-1.49	-2.59	*rplR*	LSU ribosomal protein L18P	0.133	0.007	

azo3402	-2.38	-2.45	*rplF*	LSU ribosomal protein L6P	0.022	0.040	
azo3404	-2.32	-2.66	*rpsN*	SSU ribosomal protein S14P	0.002	0.018	
azo3405	-2.95	-2.51	*rplE*	LSU ribosomal protein L5P	0.015	0.131	
azo3406	-2.54	-1.83	*rplX*	LSU ribosomal protein L24P	0.010	0.007	
azo3408	-1.99	-2.30	*rpsQ*	SSU ribosomal protein S17P	0.065	0.014	
azo3409	-1.51	-2.33	*rpmC*	LSU ribosomal protein L29P	0.045	0.002	
azo3410	-1.34	-2.07	*rplP*	LSU ribosomal protein L16P	0.074	0.020	
azo3411	-1.93	-2.89	*rpsC*	SSU ribosomal protein S3P	0.010	0.136	
azo3412	-2.62	-3.50	*rplV*	LSU ribosomal protein L22P	0.001	0.008	
azo3413	-2.49	-2.67	*rpsS*	SSU ribosomal protein S19P	0.012	0.019	
azo3414	-1.87	-3.73	*rplB*	LSU ribosomal protein L2P	0.015	0.006	
azo3415	-3.01	-2.86	*rplW*	LSU ribosomal protein L23P	0.001	0.008	
azo3416	-2.56	-3.18	*rplD*	LSU ribosomal protein L4P	0.001	0.003	
azo3417	-2.30	-2.98	*rplC*	LSU ribosomal protein L3P	0.009	0.054	
azo3418	-1.55	-2.14	*rpsJ*	SSU ribosomal protein S10P	0.038	0.016	
azo3419*	-2.05	-1.69	*tufA*	elongation factor Tu	0.017	0.001	
azo3422	-1.65	-3.66	*rpsL*	SSU ribosomal protein S12P	0.036	0.014	
azo3425	-1.71	-3.24	*rplL*	LSU ribosomal protein L12P	0.048	0.002	
azo3426	-2.29	-2.36	*rplJ*	LSU ribosomal protein L10P	0.007	0.002	
azo3428	-2.71	-3.45	*rplK*	LSU ribosomal protein L11P	0.010	0.007	
azo3429	-1.52	-2.07	*nusG*	transcription antitermination protein nusG	0.016	0.008	
azo3431	-1.84	-1.92	*tufB*	translation elongation factor 1A (EF-1A/EF-Tu)	0.002	0.004	
azo3436	1.90	-1.54	*flcA*	two component transcriptional regulator, LuxR family	0.005	0.007	
azo3442	1.88	2.39	*livF*	amino acid/amide ABC transporter ATP-binding protein 2, HAAT family	0.109	0.009	
azo3461	1.92	1.27	*pyrR*	phosphoribosyl transferase	0.011	0.007	
azo3462	1.91	1.19	*pyrB*	aspartate carbamoyltransferase	0.014	0.113	
azo3479	2.18	2.53	*fdhC*	formate dehydrogenase gamma subunit	0.183	0.010	
azo3485	4.37	15.48		conserved hypothetical iron-sulfur cluster-binding protein	0.077	0.004	
azo3486	-2.61	-1.13		conserved hypothetical protein	0.010	0.077	
azo3498	1.96	2.09		histidine kinase	0.030	0.011	
azo3522	-1.73	-3.09	*rhlE3*	putative ATP-dependent RNA helicase	0.108	0.001	
azo3528	-1.56	-2.16		conserved hypothetical membrane protein	0.009	0.016	
azo3529	-1.68	-2.34		precorrin-4 C11-methyltransferase	0.197	0.007	
azo3530	-1.49	-2.65	*cbiX*	conserved hypothetical protein cbiX	0.009	0.003	
azo3570	1.85	1.51	*waaP3*	lipopolysaccharide core biosynthesis protein	0.010	0.009	
azo3657	1.47	2.35		conserved hypothetical membrane protein	0.210	0.020	
azo3668	2.15	2.12		conserved hypothetical membrane protein	0.045	0.008	
azo3674	4.95	4.27	*mucD4*	probable serine protease MucD	0.001	0.004	down
azo3680	1.31	2.73		conserved hypothetical protein	0.252	0.001	
azo3700	1.49	2.64	*trxC2*	probable thioredoxin-disulfide reductase	0.028	0.001	
azo3758	-1.28	-2.19	*dcrH3*	putative hemerythrin-like protein	0.140	0.015	
azo3770	1.15	3.72		conserved hypothetical protein	0.244	0.007	
azo3772	1.16	2.20		conserved hypothetical protein	0.263	0.008	
azo3784	1.08	1.86		hypothetical secreted protein	0.017	0.008	
azo3790	1.33	2.14		conserved hypothetical glutathione peroxidase	0.228	0.003	
azo3815	-1.02	-2.32		probable phasin	0.047	0.018	up
azo3868	10.45	2.92	*acoB2*	probable acetoin dehydrogenase, beta subunit	0.016	0.008	
azo3872	3.19	1.72		conserved hypothetical secreted protein	0.015	0.001	
azo3873	4.11	2.21		putative glucase dehydrogenase alpha subunit	0.017	0.010	
azo3874	5.08	2.00		conserved hypothetical secreted protein	0.147	0.014	
azo3896*	1.90	1.32	*sciI*	putative cytoplasmic protein	0.024	0.015	
azo3906	1.92	1.64		hypothetical protein	0.011	0.026	
azo3911	1.94	1.27	*fabF2*	3-oxoacyl-[acyl-carrier-protein] synthase	0.015	0.022	

azo3989	-1.01	-2.01		protein translocase subunit yidC	0.180	0.022
azo3991	-1.49	-2.09		ribonuclease P protein component	0.167	0.004
azo3992	-1.95	-2.35	*rpmH*	LSU ribosomal protein L34P	0.004	0.009

[a)] Acc. No. = accession number/locus tag of *Azoarcus* sp. BH72 genome
[b)] fold change: expression ratios for each gene were calculated by dividing the corresponding intensity values from one condition by the intensity values from the other condition
to obtain an average expression fold and a respective P-value the mean of the replicates was calculated, statistical significance was determined by an one-tailed paired t-test
[c)] Proteomic studies revealed that several proteins that were differentially synthesized in a PilR regulatory mutant BH*pilR*K (Hauberg et al. 2010) were also affected under quorum sensing conditions detected by microarray at gene level. In addition, the proteins Azo0082, Azo2972, Azo3136, Azo3544 and Azo3832 were regulated under QS-conditions as well as in the *pilR*-mutant on protein level.
* also cell-density regulated on protein level as obtained by 2D-gel electrophoresis
** the gene *pilB* was not differentially expressed in the microarray approach
