# Supplementary material for: Environmental Factors Affecting the Expression of pilAB as Well as the Proteome and Transcriptome of the Grass Endophyte Azoarcus sp. Strain BH72
Source: PLoS One. 2012 Jan 20;7(1):e30421. doi: 10.1371/journal.pone.0030421 (PMC3262810; doi:10.1371/journal.pone.0030421)
Supplement: Table S4 — Detailed parameters and mass spectrometry data for proteins in Azoarcus sp. BH72 differentially synthesized upon incubation in conditioned supernatant, as discovered by MALDI-TOF-MS. (PDF) [file pone.0030421.s004.pdf]

**Table S4. Detailed parameters and mass spectrometry data for proteins in *Azoarcus* sp. BH72 differentially synthesized upon incubation in conditioned supernatant, as discovered by MALDI-TOF-MS.**

| Acc. No.[a)] | Protein | Fold | Score | | Peptide | exp.[b)] | | the.[c)] | SP[d)] | GRAVY[e)] | PSL[f)] |
|---|---|---|---|---|---|---|---|---|---|---|---|
| | | | Protein | Ion | Match | Mr (Da) | pI | Mr (Da) | | | |
| azo0082 | RdgC | -3.7 | 429 | 321 | 16 | 43448 | 4.7 | 36216 | 0 | -0.258 | C |
| azo0086* | Efp | -9.1 | 446 | 318 | 15 | 29343 | 4.6 | 20730 | 0 | -0.322 | C |
| azo0156* | AtpH | -4.6 | 120 | 56 | 9 | 18792 | 4.2 | 18803 | 0 | 0.121 | C |
| azo0174 | MreB | -6.9 | 580 | 347 | 26 | 36938 | 5.0 | 36659 | 0 | 0.146 | C |
| azo0503 | RpoX | -8.1 | 327 | 215 | 11 | 12124 | 6.4 | 12132 | 0 | -0.486 | C |
| azo0632 | IlvD | 3.2 | 167 | | 20 | 70000 | 5.5 | 65693 | 0 | -0.073 | C |
| azo0718* | RpsF | -7.6 | 420 | 319 | 12 | 14455 | 7.7 | 14464 | 0 | -0.536 | C |
| azo0721 | RplI | -3.8 | 261 | 205 | 9 | 16299 | 5.3 | 16308 | 0 | -0.126 | C |
| azo0754* | RplY | -2.9 | 630 | 450 | 19 | 21458 | 6.0 | 21471 | 0 | -0.170 | U |
| azo0769 | AhpC | -2.5 | 360 | 272 | 11 | 27771 | 5.7 | 20595 | 0 | -0.150 | C |
| azo0960 | PetA1 | -15.8 | 68 | 16 | 7 | 26908 | 6.5 | 20987 | 1 | -0.253 | CM |
| azo0963 | SspA2 | -5.2 | 234 | 175 | 9 | 26025 | 5.9 | 23164 | 0 | -0.249 | C |
| azo0973 | GroES1 | -5.2 | 248 | 140 | 10 | 15832 | 5.2 | 10411 | 0 | -0.194 | C |
| azo1042 | Asd | 3.1 | 239 | 169 | 11 | 45002 | 6.0 | 40451 | 0 | 0.055 | U |
| azo1062* | DnaJ1 | -7.9 | 367 | 296 | 15 | 49498 | 6.8 | 40839 | 0 | -0.696 | C |
| azo1063 | DnaK | -2.7 | 921 | 749 | 26 | 68718 | 4.7 | 68760 | 0 | -0.351 | C |
| azo1084 | PheT | -3.0 | 582 | 336 | 35 | 86215 | 4.9 | 85860 | 0 | 0.077 | C |
| azo1096 | DapA | -3.0 | 601 | 496 | 14 | 31860 | 5.6 | 30960 | 0 | 0.217 | U |
| azo1280* | Fpr1 | -7.5 | 724 | 488 | 24 | 29524 | 5.6 | 29165 | 0 | -0.293 | U |
| azo1358 | Ppa | -3.5 | 140 | 43 | 12 | 18714 | 4.6 | 19182 | 0 | -0.052 | C |
| azo1381 | CarA | -5.0 | 172 | 115 | 11 | 45545 | 6.0 | 40432 | 0 | 0.051 | U |
| azo1554 | GltA | -4.2 | 313 | 176 | 22 | 46998 | 6.1 | 48295 | 0 | -0.226 | C |
| azo2062* | | -2.8 | 331 | 308 | 4 | 28525 | 4.5 | 19560 | 0 | -0.235 | C |
| azo2144 | Eno | -2.7 | 761 | 614 | 21 | 45917 | 4.5 | 45945 | 0 | -0.102 | C |
| azo2146 | PyrG | 5.1 | 398 | 237 | 25 | 65682 | 5.8 | 63193 | 0 | -0.170 | C |
| azo2167 | PpsA1 | 4.7 | 577 | 366 | 33 | 83498 | 4.9 | 103784 | 0 | -0.097 | C |
| azo2396* | | -3.3 | 163 | | 18 | 79000 | 6.1 | 80001 | 0 | -0.28 | U |
| azo2414 | AcsB | 24.3 | 432 | 283 | 25 | 71528 | 5.7 | 71572 | 0 | -0.170 | C |
| azo2778 | Pgm | -3.0 | 623 | 326 | 33 | 56483 | 5.0 | 49840 | 0 | -0.101 | U |
| azo2817 | BioB | -5.2 | 301 | 243 | 11 | 35384 | 5.0 | 36900 | 0 | -0.173 | U |
| azo2934 | | -3.6 | 675 | 439 | 25 | 32719 | 6.1 | 30860 | 1 | -0.018 | U |
| azo2939 | AldA | 2.8 | 116 | 19 | 17 | 55086 | 6.0 | 55120 | 0 | -0.032 | C |
| azo2972 | ExaA2 | 3.3 | 504 | 310 | 28 | 67939 | 8.4 | 67980 | 1 | -0.437 | P |
| azo2975 | ExaA3 | 3.7 | 494 | 350 | 23 | 65000 | 8.9 | 64149 | 1 | -0.291 | P |
| azo2978 | | -4.3 | 647 | 425 | 33 | 62391 | 5.2 | 86888 | 1 | -0.432 | OM |
| azo3036 | | -3.2 | 544 | 496 | 10 | 35796 | 4.9 | 39983 | 0 | 0.078 | U |
| azo3136 | PurM | -3.4 | 633 | 529 | 15 | 42216 | 5.0 | 39223 | 0 | 0.154 | U |
| azo3419* | Tuf | -3.3 | 92 | | 8 | 47048 | 5.2 | 43116 | 0 | -0.148 | C |
| azo3419* | Tuf | -2.8 | 524 | 454 | 13 | 44783 | 5.2 | 43116 | 0 | -0.148 | C |
| azo3544 | Cat2 | 6.5 | 571 | 399 | 22 | 51471 | 6.2 | 46381 | 0 | -0.060 | U |
| azo3637 | GlmU | -4.0 | 496 | 305 | 25 | 54890 | 6.2 | 48486 | 0 | -0.054 | C |
| azo3638 | Pta | -2.9 | 245 | 148 | 17 | 58711 | 5.8 | 50411 | 0 | 0.095 | C |
| azo3832 | | -3.0 | 508 | 308 | 22 | 31000 | 9.2 | 28647 | 1 | 0.080 | U |
| azo3896* | SciI | -3.4 | 838 | 639 | 25 | 64787 | 5.0 | 55618 | 0 | -0.457 | U |

[a)] Acc. No. = accession number/locus tag of *Azoarcus* sp. BH72 genome

[b)] exp. Mr (Da) = experimental molecular mass in Dalton

[c)] the. Mr (Da) = theoretical molecular mass in Dalton and isoelectric point

[d)] The index for grand average of hydropathicity (GRAVY) was calculated with ProtParam (http://www.expasy.ch/tools/protparam.html)

[e)] The presence of signal peptides (SP) was predicted with SignalP 3.0 (Emanuelsson et al. 2007)

[f)] The subcellular localization (PSL) was predicted with PSORTb v.2 (Gardy et al. 2005)

C = Cytoplasm, CM = Cytoplasmic membrane, OM = Outer membrane, P = Periplasm, U = Unknown

* also cell-density regulated on gene expression level as obtained by microarray approach
