# Supplementary material for: Environmental Factors Affecting the Expression of pilAB as Well as the Proteome and Transcriptome of the Grass Endophyte Azoarcus sp. Strain BH72
Source: PLoS One. 2012 Jan 20;7(1):e30421. doi: 10.1371/journal.pone.0030421 (PMC3262810; doi:10.1371/journal.pone.0030421)
Supplement: Table S5 — List of differentially regulated proteins and genes of Azoarcus sp. BH72 and their corresponding COG category. (PDF) [file pone.0030421.s005.pdf]

Table S5. List of differentially regulated proteins and genes of *Azoarcus* sp. BH72 and their corresponding COG category.

Acc. No.[a)]	Gene	Product Name	COG Categories[b)]	COGs[c)]	Enzymes[d)]	Pfams[e)]	TIGRfams[f)]
azo0018		ThiJ/PfpI family protein	R	COG0693	EC:3.2.-	pfam01965	TIGR01382
azo0086	*efp*	translation elongation factor P (EF-P)	J	COG0231		pfam01132<br>pfam08207<br>pfam09285	TIGR00038
azo0092	*mucD1*	probable serine protease	O	COG0265		pfam00089<br>pfam00595	TIGR02037
azo0099	*def1*	peptide deformylase	J	COG0242		pfam01327	TIGR00079
azo0119		amino acid/amide ABC transporter substrate-binding protein, HAAT family	E	COG0683			
azo0152		conserved hypothetical membrane protein	no			pfam03899	
azo0153	*atpB*	probable ATP synthase A chain	C	COG0356	EC:3.6.3.14	pfam00119	TIGR01131
azo0154	*atpE*	ATP synthase F0 subcomplex C subunit	C	COG0636	EC:3.6.3.14	pfam00137	TIGR01260
azo0155	*atpF*	ATP synthase F0 subcomplex B subunit	C	COG0711	EC:3.6.3.14	pfam00430	TIGR01144
azo0156	*atpH*	ATP synthase F1 subcomplex delta subunit	C	COG0712	EC:3.6.3.14	pfam00213	TIGR01145
azo0157	*atpA*	ATP synthase F1 subcomplex alpha subunit	C	COG0056	EC:3.6.3.14	pfam00006<br>pfam00306<br>pfam02874	TIGR00962
azo0158	*atpG*	ATP synthase F1 subcomplex gamma subunit	C	COG0224	EC:3.6.3.14	pfam00231	TIGR01146
azo0159	*atpD*	ATP synthase beta chain	C	COG0055	EC:3.6.3.14	pfam00006<br>pfam00306<br>pfam02874	TIGR01039
azo0160	*atpC*	ATP synthase F1 subcomplex epsilon subunit	C	COG0355	EC:3.6.3.14	pfam00401<br>pfam02823	TIGR01216
azo0163		conserved hypothetical protein	S	COG3536		pfam06155	
azo0165	*phoR*	PAS/PAC sensor signal transduction histidine kinase	T	COG5002	EC:2.7.13.3	pfam00512<br>pfam02518<br>pfam11808	TIGR02966
azo0247	*bfr1*	putative bacterioferritin	P	COG2193		pfam00210	TIGR00754
azo0275		conserved hypothetical secreted protein	no				
azo0294		putative penicillin-binding protein	V	COG1680		pfam00144	

azo0300	*paaG1*	probable enoyl-CoA hydratase	I	COG1024	EC:4.2.1.17	pfam00378	TIGR02280
azo0303	*paaK*	phenylacetate-CoA ligase	H	COG1541	EC:6.2.1.30	pfam00501	TIGR02155
azo0304	*paaA*	phenylacetic acid degradation protein	S	COG3396		pfam05138	TIGR02156
azo0305	*paaB*	phenylacetic acid degradation protein	Q	COG3460		pfam06243	TIGR02157
azo0307	*paaD*	probable phenylacetic acid degradation protein	R	COG2151		pfam01883	TIGR02159
azo0308	*paaE*	probable phenylacetic acid degradation NADH oxidoreductase	C C	COG0633 COG1018		pfam00111 pfam00175 pfam00970	TIGR02160
azo0321	*cutA2*	putative protein disulfide-isomerase	C O	COG4232	EC:1.8.1.8	pfam00085 pfam02683 pfam11412	
azo0347		hypothetical secreted protein	no				
azo0352		conserved hypothetical protein	S	COG4456			
azo0368	*mmpI*	probable immunodominant 35kDa protein	no				
azo0386		hypothetical membrane protein	no				
azo0391		putative MerR-family transcriptional regulator	K	COG0789		pfam00376 pfam09278	
azo0430	*cysH*	phosphoadenylylsulfate reductase (thioredoxin)	H E	COG0175	EC:1.8.4.8	pfam01507	TIGR00434 TIGR02055
azo0432	*cysI*	putative sulfite reductase	P	COG0155	EC:1.8.1.2	pfam01077 pfam03460	
azo0438	*ansB2*	periplasmic L-asparaginase II	E J	COG0252	EC:3.5.1.1	pfam00710	TIGR00520
azo0440	*glnM*	amino acid ABC transporter membrane protein 2, PAAT family	E	COG0765		pfam00528	TIGR01726
azo0456		hypothetical secreted protein	no				
azo0457	*ragA*	two component transcriptional regulator, winged helix family	T K	COG0745		pfam00072 pfam00486	
azo0488		conserved hypothetical protein	no				
azo0499	*pheC*	amino acid ABC transporter substrate-binding protein, PAAT family	T E	COG0834		pfam00497	
azo0536		conserved hypothetical protein	no				
azo0544		hypothetical protein	no				
azo0586		Hypothetical protein	no				

azo0587	*acyH*	adenosylhomocysteinase	H	COG0499	EC:3.3.1.1	pfam00670<br>pfam05221	TIGR00936
azo0590	*htrB*	putative lipid A biosynthesis lauroyl acyltransferase	M	COG1560	EC:2.3.1.-	pfam03279	
azo0616	*bfr2*	putative bacterioferritin	P	COG2193		pfam00210	TIGR00754
azo0617	*bfd*	conserved hypothetical bacterioferritin-associated ferredoxin	P	COG2906		pfam04324	
azo0622		diguanylate cyclase/phosphodiesterase	T	COG5001		pfam00563<br>pfam00672<br>pfam00990<br>pfam08447	TIGR00229<br>TIGR00254
azo0644		putative regulatory protein	P	COG0735		pfam01475	
azo0649		conserved hypothetical protein	no				
azo0650		conserved hypothetical glycosyltransferase	M	COG0438		pfam00534<br>pfam12000	
azo0659	*gor*	NADPH-glutathione reductase	C	COG1249	EC:1.8.1.7	pfam00070<br>pfam02852<br>pfam07992	TIGR01421
azo0669	*napC1*	periplasmic nitrate reductase subunit	C	COG3005		pfam03264	TIGR02161
azo0670	*napB1*	periplasmic nitrate reductase subunit	C	COG3043		pfam03892	
azo0671	*napA1*	periplasmic nitrate reductase subunit apoprotein	C	COG0243	EC:1.7.99.4	pfam00384<br>pfam01568<br>pfam04879	TIGR01409<br>TIGR01706
azo0672	*napD1*	putative NapD protein	P	COG3062		pfam03927	
azo0673	*napE*	putative periplasmic nitrate reductase accessory protein	C	COG4459		pfam06796	TIGR02973
azo0682		conserved hypothetical sodium:solute symporter	R	COG4147		pfam00474	TIGR00813
azo0717		conserved hypothetical secreted protein	no				
azo0718	*rpsF*	SSU ribosomal protein S6P	J	COG0360		pfam01250	TIGR00166
azo0719	*priB*	putative primosomal replication protein	L	COG2965		pfam00436	
azo0720	*rpsR*	SSU ribosomal protein S18P	J	COG0238		pfam01084	TIGR00165
azo0746		hypothetical membrane protein	M	COG1807			
azo0753	*pth*	peptidyl-tRNA hydrolase	J	COG0193	EC:3.1.1.29	pfam01195	TIGR00447
azo0754	*rplY*	LSU ribosomal protein L25P	J	COG1825		pfam01386	TIGR00731
azo0755	*prsA*	Ribose-phosphate diphosphokinase	E F	COG0462	EC:2.7.6.1	pfam00156	TIGR01251
azo0864	*accB*	biotin carboxyl carrier protein	I	COG0511		pfam00364	TIGR00531

azo0865	*aroQ*	3-dehydroquinate dehydratase	E	COG0757	EC:4.2.1.10	pfam01220	TIGR01088
azo0867		conserved hypothetical protein	no				
azo0875	*mraZ*	protein mraZ	S	COG2001		pfam02381	TIGR00242
azo0898		putative serine/threonine protein kinase	T K L R	COG0515	EC:2.7.11.1	pfam00069<br>pfam00672	
azo0899		hypothetical membrane protein	T	COG1716		pfam00498	
azo0923	*ndk*	nucleoside diphosphate kinase	F	COG0105	EC:2.7.4.6	pfam00334	
azo0974	*groEL1*	chaperonin	O	COG0459		pfam00118	TIGR02348
azo1062	*dnaJ1*	chaperone protein	O	COG0484		pfam00226<br>pfam00684<br>pfam01556	TIGR02349
azo1064	*grpE*	probable heat shock protein	O	COG0576		pfam01025	
azo1072	*rpsA*	SSU ribosomal protein S1P	J	COG0539		pfam00575	TIGR00717
azo1073	*ihfB*	probable integration host factor, beta-subunit	L	COG0776		pfam00216	TIGR00988
azo1080	*infC*	bacterial translation initiation factor 3 (bIF-3)	J	COG0290		pfam00707<br>pfam05198	TIGR00168
azo1081	*rpmI*	LSU ribosomal protein L35P	J	COG0291		pfam01632	TIGR00001
azo1082	*rplT*	LSU ribosomal protein L20P	J	COG0292		pfam00453	TIGR01032
azo1090	*rpoS*	RNA polymerase, sigma 38 subunit	K	COG0568		pfam00140<br>pfam04539<br>pfam04542<br>pfam04545	TIGR02394<br>TIGR02937
azo1117	*aceA*	isocitrate lyase	C	COG2224	EC:4.1.3.1	pfam00463	TIGR01346
azo1135	*rpmG*	LSU ribosomal protein L33P	J	COG0267		pfam00471	TIGR01023
azo1159	*aceB*	AceB protein	C	COG2225	EC:2.3.3.9	pfam01274	TIGR01344
azo1193	*hppD*	probable 4-hydroxyphenylpyruvate dioxygenase	E R	COG3185	EC:1.13.11.27	pfam00903	TIGR01263
azo1212		conserved hypothetical protein	no				
azo1222	*dmpM*	phenol 2-monooxygenase	no		EC:1.14.13.7	pfam02406	
azo1226	*lguL*	lactoylglutathione lyase	E	COG0346	EC:4.4.1.5	pfam00903	TIGR00068
azo1272		conserved hypothetical secreted protein	Q	COG1463		pfam02470	TIGR00996
azo1277		conserved hypothetical glutathione peroxidase	O	COG0386	EC:1.11.1.9	pfam00255	
azo1280	*fpr1*	ferredoxin-NADP+ reductase	C	COG1018	EC:1.18.1.2	pfam00175<br>pfam00970	
azo1285	*gcvP*	glycine dehydrogenase (decarboxylating)<br>alpha subunit / beta subunit	E E	COG0403<br>COG1003	EC:1.4.4.2<br>EC:1.4.4.2	pfam02347	TIGR00461

azo1286	*gcvH*	glycine cleavage system H protein	E	COG0509		pfam01597	TIGR00527
azo1345	*ccoG*	putative iron-sulfur 4Fe-4S ferredoxin transmembrane protein	C	COG0348		pfam11614	TIGR02745
azo1349		putative universal stress protein f	T	COG0589		pfam00582	
azo1350	*phbC2*	probable poly-beta-hydroxybutyrate synthase	I	COG3243	EC:2.3.1.-	pfam00561 pfam07167	TIGR01838
azo1368		conserved hypothetical protein	no				
azo1377	*folD*	5,10-methylenetetrahydrofolate dehydrogenase (NADP+) / methenyltetrahydrofolate cyclohydrolase	H	COG0190	EC:1.5.1.5 EC:3.5.4.9	pfam00763 pfam02882	
azo1396	*nuoA*	NADH dehydrogenase subunit A	C	COG0838	EC:1.6.5.3	pfam00507	
azo1397	*nuoB2*	NADH dehydrogenase subunit B	C	COG0377	EC:1.6.5.3	pfam01058	TIGR01957
azo1399	*nuoD*	NADH dehydrogenase subunit D	C	COG0649	EC:1.6.5.3	pfam00346	TIGR01962
azo1400	*nuoE*	NADH dehydrogenase subunit E	C	COG1905	EC:1.6.5.3	pfam01257	TIGR01958
azo1401	*nuoF*	NADH dehydrogenase subunit F	C	COG1894	EC:1.6.5.3	pfam01512 pfam10531 pfam10589	TIGR01959
azo1403	*nuoH*	NADH dehydrogenase subunit H	C	COG1005	EC:1.6.5.3	pfam00146	
azo1404	*nuoI*	NADH dehydrogenase subunit I	C	COG1143	EC:1.6.5.3	pfam00037	TIGR01971
azo1406	*nuoK*	NADH dehydrogenase subunit K	C	COG0713	EC:1.6.5.3	pfam00420	
azo1407	*nuoL*	NADH dehydrogenase subunit L	C P	COG1009	EC:1.6.5.3	pfam00361 pfam00662	TIGR01974
azo1408	*nuoM*	NADH dehydrogenase subunit M	C	COG1008	EC:1.6.5.3	pfam00361	TIGR01972
azo1442		putative nuclease	V	COG1403		pfam01844	
azo1468	*exbB3*	conserved hypothetical biopolymer transport protein	U	COG0811		pfam01618	
azo1497	*prfB*	bacterial peptide chain release factor 2 (bRF-2)	J	COG1186		pfam00472 pfam03462	TIGR00020
azo1521	*rpmE*	LSU ribosomal protein L31P	J	COG0254		pfam01197	TIGR00105
azo1544		diguanylate cyclase/phosphodiesterase with PAS/PAC sensor(s)	T	COG5001		pfam00563 pfam00989 pfam00990 pfam08448	TIGR00229 TIGR00254
azo1586		conserved hypothetical secreted protein	no			pfam11454	
azo1608		hypothetical protein	no				
azo1620		conserved hypothetical protein	R	COG1399		pfam02620	
azo1621	*rpmF*	LSU ribosomal protein L32P	J	COG0333		pfam01783	TIGR01031

azo1623	*fabH*	3-oxoacyl-[acyl-carrier-protein] synthase III	I	COG0332	EC:2.3.1.180	pfam08541<br>pfam08545	TIGR00747
azo1630	*algU*	RNA polymerase, sigma-24 subunit	K	COG1595		pfam04542<br>pfam08281	TIGR02937<br>TIGR02939
azo1652		conserved hypothetical protein	S	COG5319		pfam06676	
azo1654		cell division topological specificity factor MinE	D	COG0851		pfam03776	TIGR01215
azo1659	*tex*	transcription accessory protein	K	COG2183		pfam00575<br>pfam09371	TIGR00426
azo1675		putative phosphoribosyltransferase	no				
azo1684		conserved hypothetical protein	N	COG5622		pfam10116	
azo1694		conserved hypothetical protein	R	COG3218		pfam03886	
azo1699	*etfB1*	electron transfer flavoprotein, beta subunit	C	COG2086		pfam01012	
azo1700	*etfA1*	probable electron transfer flavoprotein, alpha subunit	C	COG2025		pfam00766<br>pfam01012	
azo1701		conserved hypothetical membrane protein	S	COG3235			
azo1715	*rbcR*	transcriptional regulator	K	COG0583		pfam00126<br>pfam03466	
azo1727	*treS*	trehalose synthase	G G	COG0366<br>COG3281	EC:5.4.99.16	pfam00128	TIGR02456<br>TIGR02457
azo1743		conserved hypothetical protein	E	COG3931		pfam05013	
azo1851		conserved hypothetical protein	no				
azo1864		Hypothetical protein	no			pfam10074	
azo1874	*rmlC*	dTDP-4-dehydrorhamnose 3,5-epimerase	M	COG1898	EC:5.1.3.13	pfam00908	TIGR01221
azo1875	*rmlA*	Glucose-1-phosphate thymidylyltransferase	M	COG1209	EC:2.7.7.24	pfam00483	TIGR01207
azo1877	*rmlB*	dTDP-glucose 4,6-dehydratase	M	COG1088	EC:4.2.1.46	pfam01370	TIGR01181
azo1887		conserved hypothetical protein	S	COG3022		pfam03883	
azo1896	*lpxB*	lipid-A-disaccharide synthase	M	COG0763	EC:2.4.1.182	pfam02684	TIGR00215
azo1908	*tsf*	translation elongation factor Ts (EF-Ts)	J	COG0264		pfam00627<br>pfam00889	TIGR00116
azo1909		SSU ribosomal protein S2P	J	COG0052		pfam00318	TIGR01011
azo1918		conserved hypothetical secreted protein	R	COG2358		pfam09084	TIGR02122
azo1922	*etfB2*	electron transfer flavoprotein, beta-subunit	C	COG2086		pfam01012	
azo1948	*padD*	phenylacetyl-CoA:acceptor oxidoreductase PadD subunit	R	COG3302		pfam04976	
azo1965		transcriptional regulator, AraC family	K	COG2207		pfam00165	

azo1978		conserved hypothetical protein	R	COG1878		pfam04199	
azo1995		conserved hypothetical protein	G	COG0235		pfam00596	
azo2008		[LSU ribosomal protein L3P]-glutamine N5-methyltransferase	J	COG2890	EC:2.1.1.72	pfam05175	TIGR00536 TIGR03533 TIGR03534
azo2062		conserved hypothetical peptidyl-prolyl cis-trans isomerase	O	COG1047	EC:5.2.1.8	pfam00254	
azo2063	*bcp1*	putative bacterioferritin comigratory protein	O	COG1225	EC:1.11.1.15	pfam00578	
azo2070	*clpX*	ATP-dependent Clp protease ATP-binding subunit	O	COG1219		pfam06689 pfam07724 pfam10431	TIGR00382
azo2072	*tig*	trigger factor	O	COG0544		pfam00254 pfam05697 pfam05698	TIGR00115
azo2073	*prkA*	putative serine protein kinase	T	COG2766		pfam06798 pfam08298	
azo2074	*yeaH*	conserved hypothetical protein	S	COG2718		pfam04285	
azo2075	*ycgB*	putative cytoplasmic protein	S	COG2719		pfam04293	
azo2103	*pnp*	polyribonucleotide nucleotidyltransferase	J	COG1185	EC:2.7.7.8	pfam00013 pfam00575 pfam01138 pfam03725 pfam03726	TIGR03591
azo2104	*rpsO*	SSU ribosomal protein S15P	J	COG0184		pfam00312	TIGR00952
azo2109		conserved hypothetical protein	S	COG0779		pfam02576	
azo2120	*ylqF*	probable GTPase	R	COG1161		pfam01926	TIGR03596
azo2121	*cspA*	cold-shock DNA-binding protein family	K	COG1278		pfam00313	
azo2142		putative inosine-5'-monophosphate dehydrogenase related protein	T	COG2905		pfam00571	
azo2148	*sndH*	putative L-sorbosone dehydrogenase	G	COG2133		pfam07995 pfam12303	
azo2151	*etf1*	probable electron transfer flavoprotein-ubiquinone oxidoreductase	C C	COG0644 COG2440	EC:1.5.5.1	pfam05187	
azo2156		probable TonB-dependent receptor	P	COG1629		pfam00593 pfam07715	

azo2175	*pilY1A*	putative type 4 pilus biogenesis protein	N U	COG3419			
azo2177	*pilW*	putative type 4 pilus biogenesis protein	N U	COG4966			TIGR02532
azo2180		conserved hypothetical prepilin like protein	N U	COG4968		pfam07963	TIGR02532
azo2186	*argG*	argininosuccinate synthase	E	COG0137	EC:6.3.4.5	pfam00764	TIGR00032
azo2190	*rpsT*	SSU ribosomal protein S20P	J	COG0268		pfam01649	TIGR00029
azo2192		Hypothetical protein	no				
azo2197	*bcp2*	putative bacterioferritin comigratory protein	O	COG1225	EC:1.11.1.15	pfam00578	
azo2220	*cbiM*	putative cobalt transport system, permease protein	P	COG0310		pfam01891	
azo2224	*yaiI*	YaiI/YqxD family protein	S	COG1671		pfam02639	
azo2257	*mauA*	probable methylamine dehydrogenase, L chain	no		EC:1.4.99.3	pfam02975	TIGR02659
azo2290		conserved hypothetical protein	R	COG0121		pfam00310	
azo2314		glycosyltransferase	M	COG1807		pfam02366	
azo2324		putative polysaccharide deacetylase	G	COG0726		pfam01522	
azo2396		putative TonB-dependent receptor	P	COG1629		pfam00593<br>pfam07715	
azo2405	*ohr*	probable organic hydroperoxide resistance protein	O	COG1764		pfam02566	TIGR03561
azo2408		hypothetical sensor protein	T	COG3706		pfam00990<br>pfam11845	TIGR00229<br>TIGR00254
azo2442	*poxC*	phenol 2-monooxygenase P2 subunit	no			pfam02406	
azo2469		conserved hypothetical protein	R	COG3607			
azo2492	*etfB3*	electron transfer flavoprotein, beta subunit	C	COG2086		pfam01012	
azo2552	*pilU1*	twitching motility protein	U N	COG5008		pfam00437	TIGR01420
azo2561		conserved hypothetical protein	R	COG2823		pfam04972	
azo2563		conserved hypothetical secreted protein	S	COG4315		pfam03640	
azo2588	*ompA1*	outer membrane protein A precursor	M	COG2885		pfam00691	
azo2640	*mdcH*	putative transcriptional factor	K	COG2378			
azo2646		conserved hypothetical protein	no				
azo2651		conserved hypothetical protein	no				
azo2656		conserved hypothetical membrane protein	S	COG1742		pfam02694	
azo2664		conserved hypothetical protein	no				
azo2672		conserved hypothetical sensor histidine kinase	T	COG4191	EC:2.7.13.1	pfam00512<br>pfam02518	
azo2690		conserved hypothetical protein	S	COG3597		pfam05099	
azo2694		nucleotide sugar aminotransferase	M	COG0399		pfam01041	

azo2698	*pepM*	putative phosphoenolpyruvate phosphomutase	I M G	COG0615<br>COG2513	EC:5.4.2.9	pfam01467	TIGR00125<br>TIGR02320
azo2758		conserved hypothetical protein	S	COG0316		pfam01521	TIGR00049
azo2759	*rpsI*	SSU ribosomal protein S9P	J	COG0103		pfam00380	
azo2760	*rplM*	LSU ribosomal protein L13P	J	COG0102		pfam00572	TIGR01066
azo2763		conserved hypothetical secreted protein	no			pfam08750	
azo2790		conserved hypothetical protein	R	COG2041		pfam00174	
azo2813		conserved hypothetical secreted protein	S	COG3807		pfam06347	
azo2827	*phoU*	phosphate uptake regulator	P	COG0704		pfam01895	TIGR02135
azo2835	*prkB*	probable phosphoribulokinase	C	COG3954	EC:2.7.1.19	pfam00485	
azo2844	*exaA1*	putative quinoprotein ethanol dehydrogenase	G	COG4993	EC:1.1.99.8	pfam01011<br>pfam10527<br>pfam10535	TIGR03075
azo2845	*qbdB2*	conserved hypothetical secreted protein	C	COG4313			
azo2871		conserved hypothetical YceI like protein	S	COG2353		pfam04264	
azo2876		conserved hypothetical membrane protein	S	COG5473		pfam09955	
azo2883		conserved hypothetical secreted protein	no			pfam11127	
azo2898	*rpsP*	SSU ribosomal protein S16P	J	COG0228		pfam00886	TIGR00002
azo2901	*rplS*	LSU ribosomal protein L19P	J	COG0335		pfam01245	TIGR01024
azo2914	*pilV*	putative prepilin-like protein	N U	COG4967			TIGR02523<br>TIGR02532
azo2916	*pilX*	putative Tfp pilus assembly protein	N U	COG4726			
azo2956		putative cooper-transporting ATPase protein	P S	COG2217<br>COG3350	EC:3.6.3.4	pfam00122<br>pfam00702<br>pfam04945	TIGR01494<br>TIGR01511<br>TIGR01525
azo2969		conserved hypothetical secreted protein	no				
azo2977	*cphA*	putative beta lactamase precursor	R	COG0491		pfam00753	
azo2987		dihydrofolate reductase, putative	H	COG0262		pfam01872	
azo3023		probable TonB-dependent receptor	P	COG1629		pfam00593<br>pfam07715	
azo3041	*rubA*	probable rubredoxin	C	COG1773		pfam00301	
azo3047	*livG1*	amino acid/amide ABC transporter ATP-binding protein 1, HAAT family	E	COG0411		pfam00005<br>pfam12399	
azo3050	*livJ*	amino acid/amide ABC transporter substrate-binding protein, HAAT family	E	COG0683		pfam01094	

azo3060		conserved hypothetical protein	S	COG3945		pfam01814	
azo3073		hypothetical protein	K L R T	COG0515		pfam00069	
azo3128	*nikR*	transcriptional regulator, CopG family	K	COG0864		pfam01402<br>pfam08753	TIGR02793
azo3146		conserved hypothetical protein	T	COG2905		pfam00571	
azo3160		conserved hypothetical protein	H	COG2226		pfam08241	
azo3167	*rplU*	LSU ribosomal protein L21P	J	COG0261		pfam00829	TIGR00061
azo3168	*rpmA*	LSU ribosomal protein L27P	J	COG0211		pfam01016	TIGR00062
azo3194		conserved hypothetical protein	I	COG2030		pfam01575	
azo3212	*parA3*	ParA family protein	D	COG1192		pfam01656	
azo3225	*rpsU*	SSU ribosomal protein S21P	J	COG0828		pfam01165	TIGR00030
azo3287		conserved hypothetical secreted protein	X				TIGR01409
azo3293	*senC*	SCO1/SenC family protein	R	COG1999		pfam02630	
azo3294	*coxD*	4-hydroxybenzoate octaprenyltransferase	O	COG0109	EC:2.5.1.-	pfam01040	TIGR01473
azo3300		conserved hypothetical membrane protein	no			pfam11174	
azo3303	*coxA*	probable cytochrome c oxidase, subunit I	C	COG0843	EC:1.9.3.1	pfam00115	TIGR02891
azo3304	*coxB*	conserved hypothetical cytochrome c oxidase, subunit II	C C	COG1622<br>COG2010	EC:1.9.3.1	pfam00034<br>pfam00116<br>pfam02790	TIGR02866
azo3319	*fkbP*	peptidyl-prolyl cis-trans isomerase	O	COG0545	EC:5.2.1.8	pfam00254	
azo3320	*msrA*	putative peptide methionine sulfoxide reductase	O	COG0225	EC:1.8.4.11	pfam01625	TIGR00401
azo3325	*trpE*	anthranilate synthase, component I	H E	COG0147	EC:4.1.3.27	pfam00425<br>pfam04715	TIGR00564
azo3327	*rpe*	ribulose-5-phosphate 3-epimerase	G	COG0036	EC:5.1.3.1	pfam00834	TIGR01163
azo3330		FHA-domain containing protein	T	COG1716		pfam00498	
azo3354	*pilB*	Type IV pilus assembly protein	no				
azo3355	*pilA*	tfp structural protein	U N	COG4968		pfam07963	TIGR02532
azo3356	*pilR*	Two-component response regulator	T	COG2204		pfam00072<br>pfam00158<br>pfam02954	
azo3364		glycosyl transferase	E	COG0547		pfam02885	
azo3388		hypothetical protein	no				
azo3390	*rplQ*	LSU ribosomal protein L17P	J	COG0203		pfam01196	TIGR00059

azo3391	*rpoA*	DNA-directed RNA polymerase subunit alpha	K	COG0202	EC:2.7.7.6	pfam01000<br>pfam01193<br>pfam03118	TIGR02027
azo3393	*rpsK*	SSU ribosomal protein S11P	J	COG0100		pfam00411	TIGR03632
azo3394	*rpsM*	SSU ribosomal protein S13P	J	COG0099		pfam00416	TIGR03631
azo3395	*rpmJ*	LSU ribosomal protein L36P	no			pfam00444	TIGR01022
azo3398	*rplO*	LSU ribosomal protein L15P	J	COG0200		pfam00828	TIGR01071
azo3399	*rpmD*	LSU ribosomal protein L30P	J	COG1841		pfam00327	TIGR01308
azo3400	*rpsE*	SSU ribosomal protein S5P	J	COG0098		pfam00333<br>pfam03719	TIGR01021
azo3401	*rplR*	LSU ribosomal protein L18P	J	COG0256		pfam00861	TIGR00060
azo3402	*rplF*	LSU ribosomal protein L6P	J	COG0097		pfam00347	TIGR03654
azo3404	*rpsN*	SSU ribosomal protein S14P	J	COG0199		pfam00253	
azo3405	*rplE*	LSU ribosomal protein L5P	J	COG0094		pfam00281<br>pfam00673	
azo3406	*rplX*	LSU ribosomal protein L24P	J	COG0198		pfam00467	TIGR01079
azo3408	*rpsQ*	SSU ribosomal protein S17P	J	COG0186		pfam00366	TIGR03635
azo3409	*rpmC*	LSU ribosomal protein L29P	J	COG0255		pfam00831	TIGR00012
azo3410	*rplP*	LSU ribosomal protein L16P	J	COG0197		pfam00252	TIGR01164
azo3411	*rpsC*	SSU ribosomal protein S3P	J	COG0092		pfam00189<br>pfam00417<br>pfam07650	TIGR01009
azo3412	*rplV*	LSU ribosomal protein L22P	J	COG0091		pfam00237	TIGR01044
azo3413	*rpsS*	SSU ribosomal protein S19P	J	COG0185		pfam00203	TIGR01050
azo3414	*rplB*	LSU ribosomal protein L2P	J	COG0090		pfam00181<br>pfam03947	TIGR01171
azo3415	*rplW*	LSU ribosomal protein L23P	J	COG0089		pfam00276	
azo3416	*rplD*	LSU ribosomal protein L4P	J	COG0088		pfam00573	
azo3417	*rplC*	LSU ribosomal protein L3P	J	COG0087		pfam00297	TIGR03625
azo3418	*rpsJ*	SSU ribosomal protein S10P	J	COG0051		pfam00338	TIGR01049
azo3419	*tufA*	elongation factor Tu	J	COG0050	EC:3.6.5.3	pfam00009<br>pfam03143<br>pfam03144	TIGR00231<br>TIGR00485
azo3422	*rpsL*	SSU ribosomal protein S12P	J	COG0048		pfam00164	TIGR00981
azo3425	*rplL*	LSU ribosomal protein L12P	J	COG0222		pfam00542	TIGR00855

azo3426	*rplJ*	LSU ribosomal protein L10P	J	COG0244		pfam00466	
azo3428	*rplK*	LSU ribosomal protein L11P	J	COG0080		pfam00298<br>pfam03946	TIGR01632
azo3429	*nusG*	transcription antitermination protein nusG	K	COG0250		pfam02357	TIGR00922
azo3431	*tufB*	translation elongation factor 1A (EF-1A/EF-Tu)	J	COG0050	EC:3.6.5.3	pfam00009<br>pfam03143<br>pfam03144	TIGR00231<br>TIGR00485
azo3436	*flcA*	two component transcriptional regulator,<br>LuxR family	T K	COG2197		pfam00072<br>pfam00196	
azo3442	*livF*	amino acid/amide ABC transporter<br>ATP-binding protein 2, HAAT family	E	COG0410		pfam00005	
azo3461	*pyrR*	phosphoribosyl transferase	F	COG2065	EC:2.4.2.9	pfam00156	
azo3462	*pyrB*	aspartate carbamoyltransferase	F	COG0540	EC:2.1.3.2	pfam00185<br>pfam02729	TIGR00670
azo3479	*fdhC*	formate dehydrogenase gamma subunit	C	COG2864	EC:1.2.1.2	pfam00033	TIGR01583
azo3485		conserved hypothetical iron-sulfur<br>cluster-binding protein	C	COG1149		pfam00037	
azo3486		conserved hypothetical protein	no			pfam11748	
azo3498		histidine kinase	T	COG0642		pfam02518	
azo3522	*rhlE3*	putative ATP-dependent RNA helicase	L K J	COG0513		pfam00270<br>pfam00271	
azo3528		conserved hypothetical membrane protein	P	COG0310		pfam01891	
azo3529		precorrin-4 C11-methyltransferase	H	COG2875	EC:2.1.1.133	pfam00590	TIGR01465
azo3530	*cbiX*	conserved hypothetical protein	S	COG2138	EC:4.99.1.3	pfam01903	
azo3570	*waaP3*	lipopolysaccharide core biosynthesis protein	no			pfam06293	
azo3657		conserved hypothetical membrane protein	S	COG3671			
azo3668		conserved hypothetical membrane protein	S	COG5393		pfam07332	
azo3674	*mucD4*	probable serine protease	O	COG0265		pfam00089<br>pfam00595	TIGR02037
azo3680		conserved hypothetical protein	no			pfam10932	
azo3700	*trxC2*	probable thioredoxin-disulfide reductase	O	COG3118	EC:1.8.1.9	pfam00085	TIGR01068
azo3758	*dcrH3*	putative hemerythrin-like protein	P	COG2703		pfam01814	TIGR02481
azo3770		conserved hypothetical protein	S	COG1652		pfam01476<br>pfam04972	
azo3772		conserved hypothetical protein	R	COG2081		pfam03486	TIGR00275

azo3784		hypothetical secreted protein	no				
azo3790		conserved hypothetical glutathione peroxidase	O	COG0386	EC:1.11.1.9	pfam00255	
azo3815		probable phasin	no			pfam09361	TIGR01841
azo3868	*acoB2*	probable acetoin dehydrogenase, beta subunit	C	COG0022	EC:1.2.4.1	pfam02779<br>pfam02780	
azo3872		conserved hypothetical secreted protein	no				TIGR01409
azo3873		putative glucase dehydrogenase alpha subunit	E	COG2303		pfam00732<br>pfam05199	
azo3874		conserved hypothetical secreted protein	E	COG0346		pfam00903	
azo3896	*sciI*	putative cytoplasmic protein	S	COG3517		pfam05943	TIGR03355
azo3906		hypothetical protein	no				
azo3911	*fabF2*	3-oxoacyl-[acyl-carrier-protein] synthase	I Q	COG0304	EC:2.3.1.41	pfam00109<br>pfam02801	
azo3989		protein translocase subunit yidC	U	COG0706		pfam02096	TIGR03592<br>TIGR03593
azo3991		ribonuclease P protein component	J	COG0594	EC:3.1.26.5	pfam00825	TIGR00188
azo3992	*rpmH*	LSU ribosomal protein L34P	J	COG0230		pfam00468	TIGR01030

[a)] Acc. No. = accession number/locus tag of *Azoarcus* sp. BH72 genome

[b)] COG-categories = Cluster of orthologous groups of proteins according to Tatusov *et al.* 1997, A: RNA processing and modification, B: Chromatin structure and dynamics, C: Energy production and conversion, D: Cell cycle control, mitosis and meiosis, E: Amino acid transport and metabolism, F: Nucleotide transport and metabolism, G: Carbohydrate transport and metabolism, H: Coenzyme transport and metabolism, I: Lipid transport and metabolism, J: Translation, K: Transcription, L: Replication, recombination and repair, M: Cell wall/membrane biogenesis, N: Cell motility, O: Posttranslational modification, protein turnover, chaperones, P: Inorganic ion transport and metabolism, Q: Secondary metabolites biosynthesis, transport and catabolism, R: General function prediction only, S: Function unknown, T: Signal transduction mechanisms, U: Intracellular trafficking and secretion, no: not in COG.

[c)] COG = Cluster of orthologous groups of proteins according to Tatusov *et al.* 1997

[d)] Enzymes = EC Number given by the IUBMB (International Union of Biochemistry and Molecular Biology), classes of enzymes und subclasses defined according to the reaction catalyzed, BRENDA (Chang *et al.* 2009, http://www.brendaenzymes. org/)

[e)] Pfam = Protein families according to Pfam database (Finn *et al.* 2010, http://pfam.sanger.ac.uk/)

[f)] Tigrfam = Protein families according to TIGRFAM database (Haft *et al.* 2003, http://blast.jcvi.org/web-hmm/)
