# Supplementary figures and images for: Environmental Factors Affecting the Expression of pilAB as Well as the Proteome and Transcriptome of the Grass Endophyte Azoarcus sp. Strain BH72
Source: PLoS One. 2012 Jan 20;7(1):e30421. doi: 10.1371/journal.pone.0030421 (PMC3262810; doi:10.1371/journal.pone.0030421)

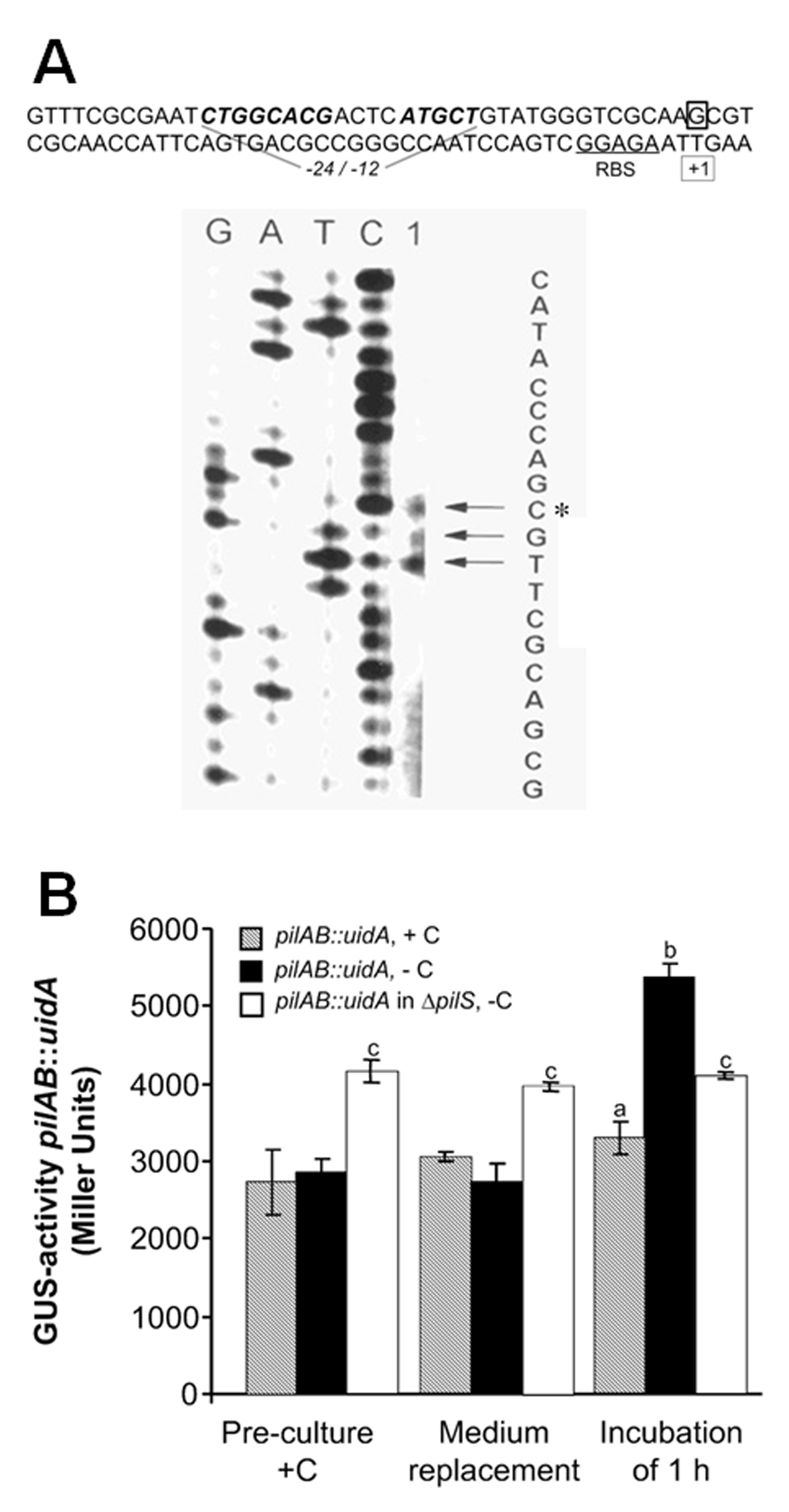

Supplement: Figure S1 — Analysis of pilAB expression. (A) Representation of the pilAB region in the chromosome of Azoarcus sp. strain BH72 and primer extension analysis. The DNA sequence upstream of the pilAB operon is labeled for a σ54-dependent promoter site (−24/−12, bold italics), the transcriptional start point (+1, small box) and the ribosome binding site (RBS, underlined). Below, primer extension analysis of the start site of the pilAB transcript. Lanes G A T C contain mixtures from DNA sequencing reactions performed with the primer complementary to the sequence of pilA 42 bp downstream of the predicted transcription start. In lane 1 the primer extension reaction from RNA extract of wild type cells was loaded, and the longest transcript labeled with a star. (B) Expression of a chromosomal pilAB::uidA fusion in wild type and ΔpilS mutant background under conditions of carbon starvation. Cells were grown in a pre-culture of complex medium (VM-Ethanol), washed two times with synthetic medium (SM) with or without the carbon source potassium malate (medium replacement), and then grown for 1 hour in the respective medium with or without carbon source (+/− C). Error bars indicate standard deviations from three replicates. Similar results were obtained in at least three independent experiments. Statistical analysis: columns labeled with different letters differ statistically significantly from each other (P<0.0001, unpaired t-test). (TIF) [file pone.0030421.s006.tif]

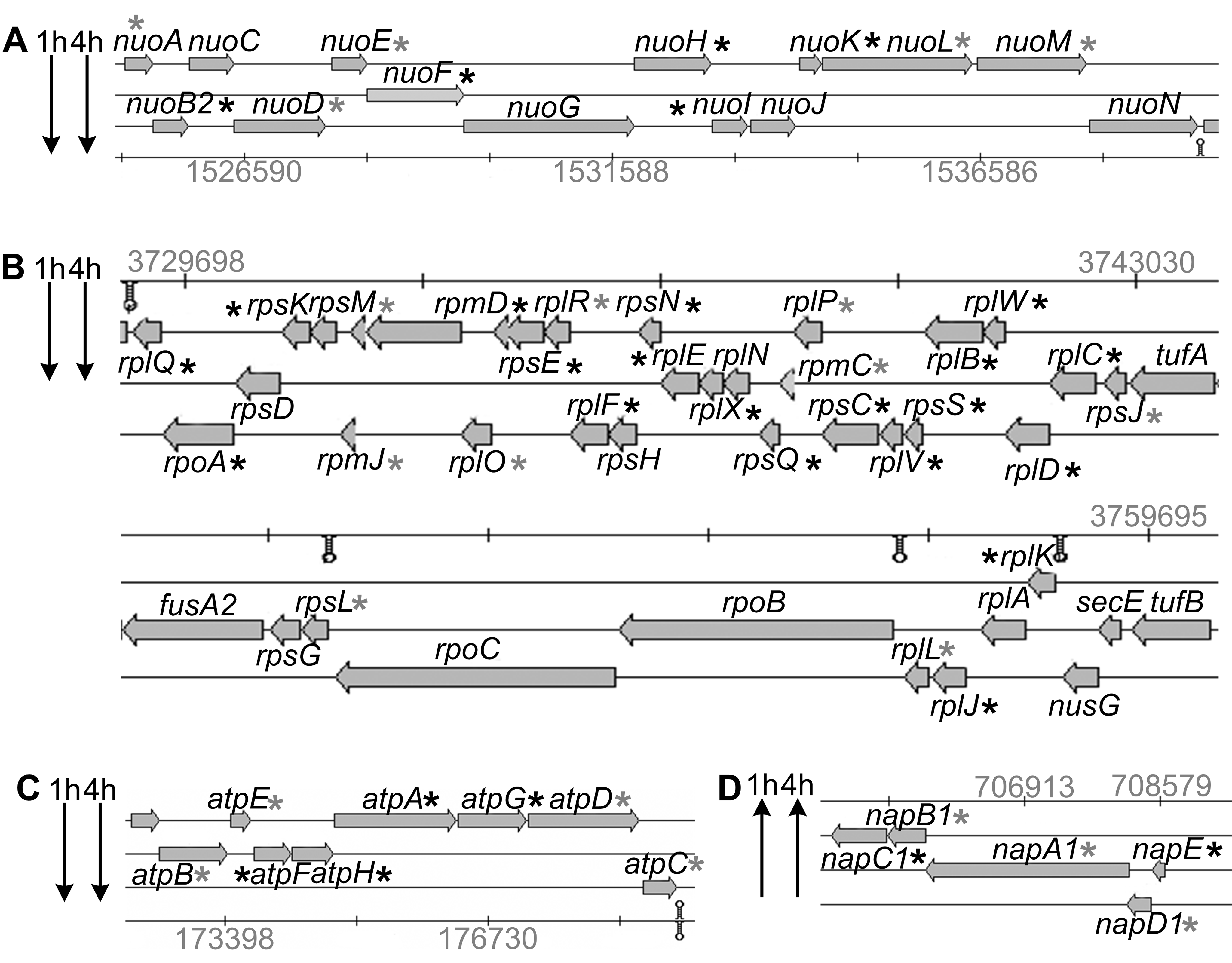

Supplement: Figure S2 — Differentially regulated gene clusters as identified by transcriptome microarray upon incubation in conditioned supernatant. (A–D), Four representative gene clusters of Azoarcus sp. BH72 are shown. Activated clusters are depicted with arrows showing upwards, and repressed clusters with arrows showing downwards, detected at the distinct time point of one or four hours of incubation, respectively. Stars indicate genes whose expression in the cluster was only changed after four hour (grey stars) or after one and four hours (black stars) of incubation with conditioned culture supernatant. Bold grey arrows refer to the direction of transcription with the respective gene name in black letters. Locations in the Azoarcus genome are depicted with grey numbers. (TIF) [file pone.0030421.s007.tif]

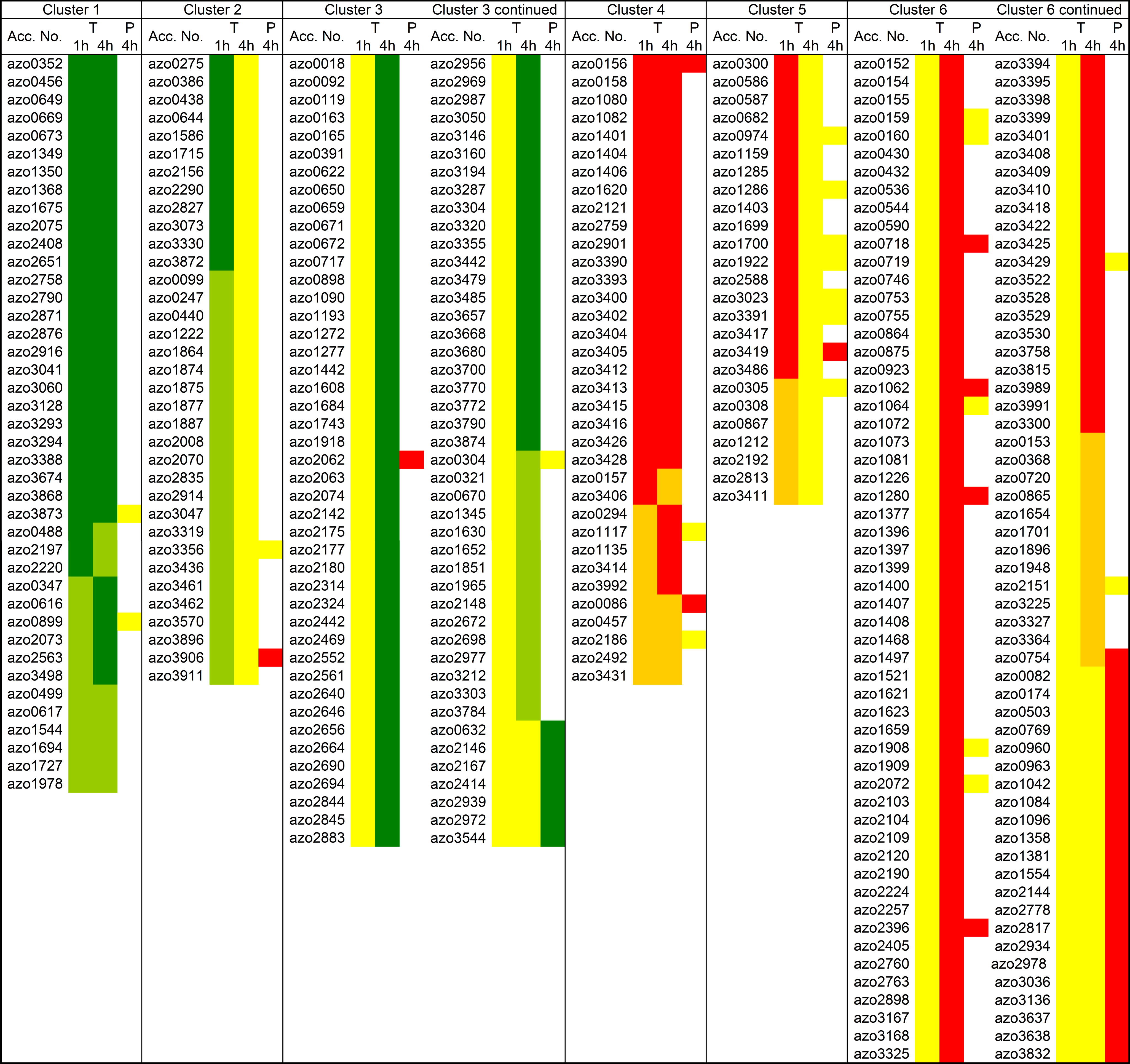

Supplement: Figure S3 — Comparison of differential regulation in Azoarcus sp. BH72 upon incubation in conditioned supernatant, revealed by transcriptomic (T) and proteomic (P) approaches. Colours in heat map indicate the fold-change in gene expression or protein synthesis, respectively, in conditioned supernatants. Colour code: light green = ≥+1.8, dark green ≥+2.0 for transcriptomic study and ≥+2.5 for proteomic study, orange ≤−1.8, red ≤−2.0 for transcriptomic study and ≤−2.5 for proteomic study, yellow = no change, white = not detected in 2D-gels). (TIF) [file pone.0030421.s008.tif]
